# Cytomegalovirus immediate-early 1 proteins form a structurally distinct protein class with adaptations determining cross-species barriers

**DOI:** 10.1371/journal.ppat.1009863

**Published:** 2021-08-09

**Authors:** Johannes Schweininger, Myriam Scherer, Franziska Rothemund, Eva-Maria Schilling, Sonja Wörz, Thomas Stamminger, Yves A. Muller

**Affiliations:** 1 Division of Biotechnology, Department of Biology, Friedrich-Alexander-University Erlangen-Nürnberg, Erlangen, Germany; 2 Institute of Virology, Ulm University Medical Center, Ulm, Germany; State University of New York Upstate Medical University, UNITED STATES

## Abstract

Restriction factors are potent antiviral proteins that constitute a first line of intracellular defense by blocking viral replication and spread. During co-evolution, however, viruses have developed antagonistic proteins to modulate or degrade the restriction factors of their host. To ensure the success of lytic replication, the herpesvirus human cytomegalovirus (HCMV) expresses the immediate-early protein IE1, which acts as an antagonist of antiviral, subnuclear structures termed PML nuclear bodies (PML-NBs). IE1 interacts directly with PML, the key protein of PML-NBs, through its core domain and disrupts the dot-like multiprotein complexes thereby abrogating the antiviral effects. Here we present the crystal structures of the human and rat cytomegalovirus core domain (IE1_CORE_). We found that IE1_CORE_ domains, also including the previously characterized IE1_CORE_ of rhesus CMV, form a distinct class of proteins that are characterized by a highly similar and unique tertiary fold and quaternary assembly. This contrasts to a marked amino acid sequence diversity suggesting that strong positive selection evolved a conserved fold, while immune selection pressure may have fostered sequence divergence of IE1. At the same time, we detected specific differences in the helix arrangements of primate versus rodent IE1_CORE_ structures. Functional characterization revealed a conserved mechanism of PML-NB disruption, however, primate and rodent IE1 proteins were only effective in cells of the natural host species but not during cross-species infection. Remarkably, we observed that expression of HCMV IE1 allows rat cytomegalovirus replication in human cells. We conclude that cytomegaloviruses have evolved a distinct protein tertiary structure of IE1 to effectively bind and inactivate an important cellular restriction factor. Furthermore, our data show that the IE1 fold has been adapted to maximize the efficacy of PML targeting in a species-specific manner and support the concept that the PML-NBs-based intrinsic defense constitutes a barrier to cross-species transmission of HCMV.

## Author summary

Cytomegaloviruses have evolved in very close association with their hosts resulting in a highly species-specific replication. Cell-intrinsic proteins, known as restriction factors, constitute important barriers for cross-species infection of viruses. All cytomegaloviruses characterized so far express an abundant immediate-early protein, termed IE1, that binds to the cellular restriction factor promyelocytic leukemia protein (PML) and antagonizes its repressive activity on viral gene expression. Here, we present the crystal structures of the PML-binding domains of rat and human cytomegalovirus IE1. Despite low amino-acid sequence identity both proteins share a highly similar and unique fold forming a distinct protein class. Functional characterization revealed a common mechanism of PML antagonization. However, we also detected that the respective IE1 proteins only interact with PML proteins of the natural host species. Interestingly, expression of HCMV IE1 allows rat cytomegalovirus infection in human cells. This indicates that the cellular restriction factor PML forms an important barrier for cross-species infection of cytomegaloviruses that might be overcome by adaptation of IE1 protein function. Our data suggest that the cytomegalovirus IE1 structure represents an evolutionary optimized protein fold targeting PML proteins via coiled-coil interactions.

## Introduction

To combat viral infections, host organisms have developed an intricate defense network comprising the intrinsic, innate, and adaptive immune response. While innate and adaptive defense mechanisms rely on pathogen-induced activation, the intrinsic immune system is conferred by constitutively expressed restriction factors thus mediating a front-line defense against invading pathogens [1]. Since the discovery of the first class of restriction factors targeting retroviral capsids, numerous cellular factors have been identified that restrict diverse steps in the life cycle of viruses [2]. During the evolutionary “arms race”, however, viruses have evolved means to evade or directly counteract these antiviral host factors, mainly by expressing antagonistic proteins. The evolutionary pressure that restriction factors and antagonists have exerted on each other resulted in further adaptations at the virus-host interface. Thus, restriction factors are often less effective against viral infections of their natural host but constitute potent barriers to cross-species infections [3].

Specific structures within the cell nucleus termed PML nuclear bodies (PML-NBs) or nuclear domain 10 (ND10) have been shown to play a major role in the intrinsic defense against a variety of viruses, including members of the highly host-adapted herpesvirus family [4]. PML-NBs are dynamic multiprotein complexes that accumulate in distinct foci within the interchromosomal space and have been implicated in cellular key processes such as cell cycle progression, apoptosis, senescence, DNA damage and antiviral responses [5]. PML, the signature protein of PML-NBs, belongs to the immunomodulatory tripartite motif (TRIM) protein family, whose members share an N-terminal domain structure comprising a RING domain, one or two B-Boxes, and a coiled-coil (CC) domain (often subsumed under the term RBCC domain) [6]. Within the N-terminal region, PML additionally harbors target sites for covalent modification with small ubiquitin-like modifier (SUMO) proteins, which enables the interaction with further protein components and, therefore, is essential for PML-NB biogenesis [7,8]. Upon herpesvirus infection, PML-NBs associate with viral genomes as soon as they have entered the nucleus [9,10]. This association blocks viral infection at a very early step, since PML-NB proteins rapidly promote the condensation of herpesviral DNA into transcriptionally inactive heterochromatin [11]. Besides PML, several other PML-NB components including Sp100, hDaxx, ATRX, and MORC3 function as restriction factors and contribute to the repression of viral gene expression in a cooperative manner [12–16].

In order to overcome the PML-NB-based defense, herpesviruses encode antagonistic effector proteins, which employ different strategies to either inactivate single PML-NB components or to disrupt the integrity of the whole structure. The herpes simplex virus type I immediate-early protein ICP0, for instance, disarms PML-NBs in a rapid and efficient way by inducing a widespread proteasomal degradation of SUMO-modified proteins including PML-NB components [17]. In contrast, immediate-early protein IE1 of human cytomegalovirus (HCMV), a ubiquitous β-herpesvirus causing serious disease in immunocompromised individuals, uses a more careful strategy, likely due to the prolonged replication cycle of HCMV. IE1 directly interacts with PML and blocks its SUMOylation in a proteasome-independent manner [18,19]. Since SUMO modification of PML is essential for PML-NB integrity, this results in a dispersal and inactivation of PML-NB foci. Structural characterization of IE1 has shown that it comprises a folded core domain (IE1_CORE_), which mediates the interaction with PML and is flanked by a short disordered region at the N-terminus and a longer disordered region at the C-terminus containing a SUMOylation motif and a STAT interaction site [20–22]. Crystallization of the IE1_CORE_ domain of rhesus cytomegalovirus (RhCMV), as described in a previous publication of our groups, revealed a so-far unobserved femur-like all-α-helical fold with local similarity to the conserved coiled-coil domain of TRIM proteins [22]. Since IE1_CORE_ efficiently binds to the PML (TRIM19) coiled-coil domain, we proposed that IE1 sequesters PML via structural mimicry using an extended binding surface.

In this study, we present the experimentally determined crystal structures of human and rat cytomegalovirus (RCMV) IE1_CORE_. All crystallized IE1_CORE_ domains share a highly similar, all-α-helical fold. Since we observed that the mechanism of PML-NB disruption is likewise conserved between primate and rodent IE1 proteins, we conclude that cytomegaloviruses have evolved this distinct protein fold to effectively bind and inactivate an important antiviral defense. Closer investigation of the crystal structures revealed slight differences in the helix arrangement of rat compared to primate cytomegalovirus IE1. This correlates with a comparative functional analysis of human and rat cytomegalovirus IE1 showing that neutralization of PML-NBs occurs only in cells of the natural host species but not during cross-species infection. For RCMV, this block of cross-species infection can be alleviated by expression of human IE1 in human host cells. In summary, our data provide evidence that the IE1 fold has been adapted to maximize the efficiency of PML-NB targeting and strengthen the concept that the PML-NBs-based intrinsic defense constitutes a barrier to cross-species transmission of HCMV.

## Results

### The domain organization of IE1 is conserved across primates and rodents

**Fig 1:**
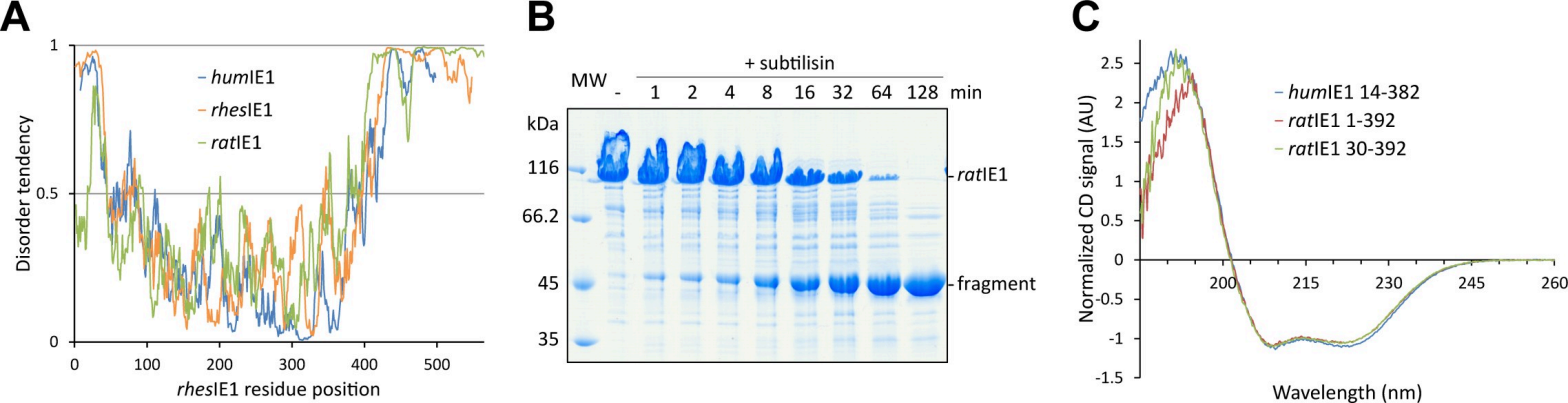
Analysis of the domain organization of *rat*IE1. (A) *In silico* disorder prediction analysis of human (*hum*), rhesus (*rhes*) and rat (*rat*) cytomegalovirus IE1 sequences using IUPred2A [69]. The disorder score for all three proteins suggest a globular domain with disordered N- and C-termini (scores ≥ 0.5 indicate disorder). (B) Limited proteolysis of recombinant *rat*IE1. Purified *rat*IE1 was incubated with subtilisin (1 mU protease per mg *rat*IE1) for different times, and samples were analyzed by SDS-PAGE and Coomassie blue staining. (C) CD spectroscopy of *hum*IE1 14–382, *rat*IE1 1–392 and *rat*IE1 30–392. The spectra were normalized at 207 nm as suggested by Raussens and coworkers [57].

The architecture of cytomegalovirus IE1 proteins appears to be evolutionary conserved across species. An *in silico* disorder prediction of the rodent member rat cytomegalovirus IE1 protein (*rat*IE1) is in agreement with the presence of a folded core domain that is flanked by a short partially or fully disordered N-terminal segment as well as a disordered extended C-terminal segment as previously observed in the primate IE1 proteins from human (*hum*IE1) and rhesus (*rhes*IE1) cytomegalovirus (Fig 1A) [22].

An experimental validation of the *in silico* prediction via a limited proteolysis digestion of full-length recombinant *rat*IE1 (residues 1 to 565) yielded a single and stable 45 kDa fragment (Fig 1B). A mass spectrometry analysis of this fragment revealed that it extends from residues 1 to 392 of the *rat*IE1 sequence (S1 Fig). It includes the very N-terminal residues of *rat*IE1, which in case of the homologous *rhes*IE1 and *hum*IE1 proteins, were prone to digestion in previous experiments and therefore postulated as not being part of the core domains in these proteins (*hum*IE1_CORE_, residues 14 to 382; *rhes*IE1_CORE_, residues 36 to 395) [22]. Interestingly, the *in silico* disorder analysis of *rat*IE1 anticipated this result since the predicted disorder tendency for the first 16 residues is considerably reduced in *rat*IE1 versus *hum*- and *rhes*IE1 (Fig 1A).

One *hum*IE1 and two *rat*IE1 variants were produced for further characterization. The two *rat*IE1 variants, covering residues 1 to 392 and residues 30 to 392, show an almost identical all-α-helical secondary structure composition as analyzed by CD spectroscopy (Fig 1C). Moreover, the CD spectra of the two *rat*IE1 variants are almost indistinguishable from that of *hum*IE1_CORE_ in agreement with the assumption of a shared core domain in IE1 proteins (Fig 1C). Of the two *rat*IE1 variants, only the N-terminally truncated variant yielded protein crystals. This variant, covering residues 30 to 392, is from here-on referred to as the *rat*IE1_CORE_ domain. Taken together, *rat*IE1, *hum*IE1 and *rhes*IE1 share an approximately 350-residue, all-α-helical core domain that is flanked by a short, fully or partially disordered region at the N-terminus and a 110- to 170-residue-long disordered region at the C-terminus.

### Crystal structures of *rat*IE1_CORE_ and *hum*IE1_CORE_

**Fig 2:**
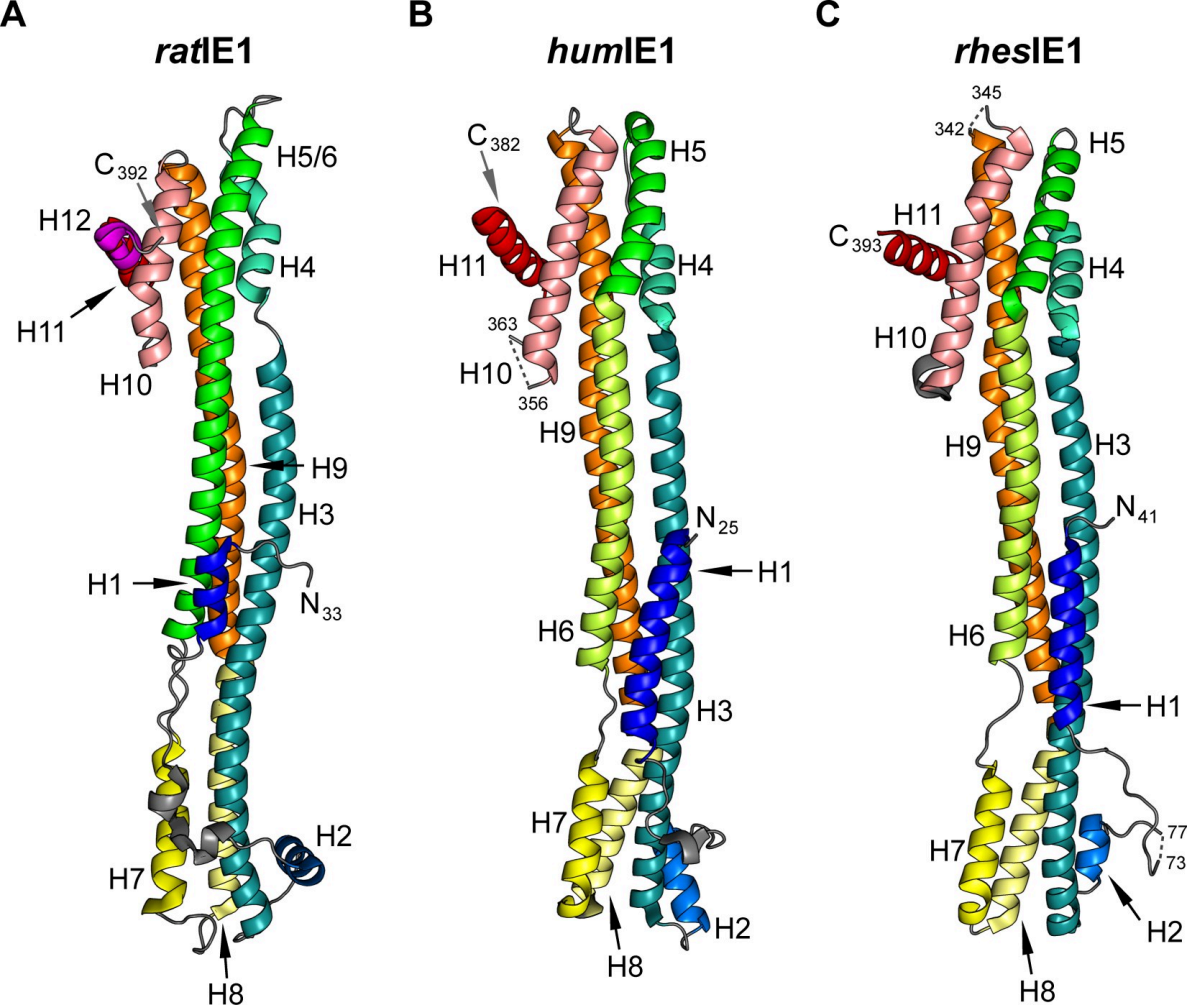
*Rat*IE1_CORE_, *hum*IE1_CORE_ and previously characterized *rhes*IE1_CORE_ share a common and unique fold. Ribbon representation of *rat*IE1 30–392 (A), *hum*IE1 14–382 (B) and *rhes*IE1 36–395 (C) (PDB: 4WID, chain B). The helices are colored from blue to pink for *rat*IE1 and from blue to red for *hum*IE1 and *rhes*IE1. N- and C-terminal residues as well as residues flanking chain breaks are labeled.

The structure of *rat*IE1_CORE_ was solved to 3.4 Å resolution with *R*_work_ = 21.5% and *R*_free_ = 24.6% (Table 1). Initial phases were obtained with the MAD technique since any molecular replacement calculations with the previously determined *rhes*IE1_CORE_ structure failed [22]. The main chain of *rat*IE1_CORE_ could be built from residues 33 to 392, and only three N-terminal residues could not be located in the electron density (Fig 2A). Because of the low resolution of the crystallographic analysis, the correctness of the sequence registration was corroborated by additional experiments. Firstly, we calculated an anomalous difference map with phases derived from the refined structure and amplitudes from the seleno-methionine peak diffraction data set (Table 1). A close inspection of this difference map showed that all eleven peaks with densities above 4.6 σ can be explained by the 12 selenium atoms present in seleno-methionine-substituted *rat*IE1_CORE_ (S1 Table). No density peaks above 3.7 σ remain unaccounted for. Secondly, we recorded a long-wavelength 6 keV X-ray diffraction data set from non-substituted *rat*IE1_CORE_ crystals in order to maximize the anomalous sulfur signal (Table 1). An anomalous difference map calculated with these data showed clear peaks for the sulfur atoms of all 7 cysteine and 10 out of 12 methionine residues present in *rat*IE1_CORE_ (S2 Table). Albeit no clear peaks were observed at the sulfur position of methionines 83 and 391 in this map, the positions of the atoms were clearly visible in the analysis of the previous seleno-methionine peak diffraction data set.

**Table 1:** Crystallographic data collection, phasing and refinement statistics.

PDB deposition code	6TH1				6TGZ
**Data collection**					
Crystal	*rat*IE1 30–392		SeMet *rat*IE1 30–392		*hum*IE1 14–382
Space group	P6_5_22				C222_1_
Cell dimensions					
*a*, *b*, *c* (Å)	173.1, 173.1, 133.5		173.4, 173.4, 133.9		110.0, 134.1, 70.5
α, β, γ (°)	90.0, 90.0, 120.0				90.0, 90.0, 90.0
Dataset	Native	6 keV^a^^,^^b^	Peak^a^^,^^c^	Inflection^a^	Native
Wavelength (Å)	0.9184	2.0664	0.9797	0.9798	2.033
Resolution (Å)					
Spherical	20–3.4 (3.6–3.4)^d^	20–5.0 (5.1–5.0)	20–4.0 (4.1–4.0)	20–4.0 (4.1–4.0)	20–3.2 (3.3–3.2)
Ellipsoidal					3.24 (a* direction) 3.29 (b* direction) 3.20 (c* direction)
*R*_meas_ (%)	35.7 (605)	11.4 (39.4)	48.2 (202)	40.2 (192)	21.9 (150)
*R*_*pim*_ (%)	3.9 (67.0)	n.d.^e^	n.d.	n.d.	6.0 (40.6)
*I/σ(I)*	19.2 (0.9)	35.0 (13.7)	14.0 (2.9)	7.3 (1.8)	8.4 (1.1)
*CC*_1/2_ (%)	100.0 (29.5)	100.0 (99.4)	99.8 (80.1)	99.6 (76.7)	99.8 (79.2)
CC* (%)	100 (67.5)				100 (94.5)
Resolution limit anomalous signal (Å)^f^	n.d.	5.0	5.0	6.0	n.d.
Completeness (%)					
Spherical	99.0 (97.2)	98.4 (100.0)	99.8 (98.5)	99.9 (100.0)	92.8 (63.6)
Ellipsoidal					95.9 (88.7)
Multiplicity	80	72	90	21	13
**Refinement**					
Resolution range (Å)	20–3.4 (3.5–3.4)				20–3.2 (3.3–3.2)
No. of unique reflections	16608				8257
Reflections used for *R*_free_	1626 (158)				800 (47)
*R*_work_ (%)	21.5 (35.4)				22.5 (30.7)
*R*_free_ (%)	24.6 (37.0)				26.8 (29.5)
*CC*_work_ (%)	97.9 (53.6)				93.8 (60.0)
*CC*_free_ (%)	96.4 (64.0)				94.9 (84.9)
Ramachandran (%)					
favored/outlier	98.0/0.0				98.3/0.0
Total no. of atoms	2799				2807
No. of protein atoms	2799				2807
*B*-factors (Å)^2^	130				54.8
No. of TLS groups	3				8
R.m.s deviations					
Bond lengths (Å)	0.004				0.005
Bond angles (°)	0.84				0.91

^a^ Values calculated with the Friedel’s law equal false setting.

^b^ Merged data from seven crystals.

^c^ Merged data from five crystals.

^d^ Values in parentheses refer to the highest resolution shell.

^e^ not determined

^f^ Defined as the resolution value where the correlation between anomalous differences drops below 30%. Values estimated from SHELXC [61].

The crystal structure of *hum*IE1_CORE_ was solved to 3.2 Å resolution with *R*_work_ = 22.5%, *R*_free_ = 26.8% (Table 1). Initial phases were obtained by molecular replacement with program MR-Rosetta and using *rhes*IE1_CORE_ as a search model [23]. In the final model, the *hum*IE1_CORE_ protein chain could be traced from residues 25 to 356 and 363 to 382 (Fig 2B).

### *Rat*IE1, *hum*IE1 and *rhes*IE1 share a unique fold

*Rat*IE1_CORE_ and *hum*IE1_CORE_ share a highly similar overall fold, which bears close resemblance to that of the previously determined *rhes*IE1_CORE_ structure (Figs 2 and S2) [22]. All three IE1_CORE_ proteins display a femur-like structure consisting of α-helices only. These are arranged into two head regions interconnected by a stalk region composed of three to four long α-helices. *Hum*IE1_CORE_ resembles *rhes*IE1_CORE_ more closely than *rat*IE1_CORE_. *Hum*IE1_CORE_ can be superimposed onto *rhes*IE1_CORE_ with an rmsd_Cα_ value of 2.3 Å, while the structures of *hum*IE1_CORE_ and *rat*IE1_CORE_ differ by an rmsd_Cα_ value of as high as 4.6 Å (Table 2). The structural deviations between these proteins are paralleled by marked differences in sequence identities. While *hum*IE1_CORE_ and *rhes*IE1_CORE_ can be aligned with 24% sequence identity, the sequence identity between *hum*IE1_CORE_ and *rat*IE1_CORE_ amounts to only 22% (Table 2).

**Table 2:** Sequence and structure similarities between IE1_CORE_ domains.

	*rat*IE1 *versus hum*IE1	*rat*IE1 *versus rhes*IE1	*hum*IE1 *versus rhes*IE1
Sequence identities (%)^a^			
Entire coding regions	23	23	27
IE1_CORE_ domains	22	22	24
Inferred from a structure-based alignment of the core domains^b^	9	9	22
Structure similarities, rmsd_Cα_ values (Å)^c^			
IE1_CORE_ domain monomers	4.6 (319)^d^	4.0 (316)	2.3 (337)
IE1_CORE_ domain dimers	4.3 (623)	4.4 (612)	2.6 (673)

^a^ Sequence identities observed in pairwise sequence alignments using Clustal Omega [76].

^b^ Derived from a pair-wise structure-based alignment calculated using DALI [70].

^c^ Root mean square deviations calculated using Cα atoms only. Calculated using DALI [70].

^d^ The number of aligned Cα positions is provided in parentheses.

It is known from comparative structural biology that the lower the sequence identities between the proteins, the more dissimilar the structures of the respective proteins are and *vice versa* [24]. However, sequence identities as low as 24 or 22% fall below the cut-off value of 28% that has been derived as a lower limit for safely inferring structural details and overall similarities from sequence identities in proteins of more than 200 residues in length [25,26]. As a consequence of the low sequence identities, *hum*IE1_CORE_, *rhes*IE1_CORE_ and *rat*IE1_CORE_ exhibit marked differences (Figs 2 and S2). Thus, helix H1 is significantly shorter in *rat*IE1_CORE_, and the position of H2 is rotated by approximately 90° in comparison to *hum*IE1_CORE_ and *rhes*IE1_CORE_. Furthermore, a kink separates helices H5 and H6 in *hum*IE1_CORE_ and *rhes*IE1_CORE_, whereas *rat*IE1_CORE_ contains one continuous helix termed H5/6. Besides this, the curvature of several helices, namely H3, H6 and H9, also slightly differs between *hum*IE1_CORE_ and *rhes*IE1_CORE_ on one hand and *rat*IE1_CORE_ on the other hand. At the same time, *rat*IE1_CORE_ has an additional helix H12 at the C-terminus in comparison to *hum*IE1_CORE_ and *rhes*IE1_CORE_, which consist of eleven helices in total.

A DALI search against the entire protein data bank (PDB, performed in February 2021) unambiguously identifies these three proteins as forming a unique structure family (S3 Table) [27,28]. Additional candidate homologous proteins, as identified by DALI, either display excessively high rmsd_Cα_ values exceeding 8 Å when aligning up to 240 residues or the structural homology is limited to considerably smaller segments of about 100 residues in the compared proteins so that rmsd_Cα_ values of about 3 Å upwards are obtained (S3 Table). This shows that clear structural homology extending over the entire length of the compared protein structures is only detectable within the group of IE1_CORE_ proteins, but not to any other protein of known structure.

### CMV IE1 proteins display an identical dimerization mode

**Fig 3:**
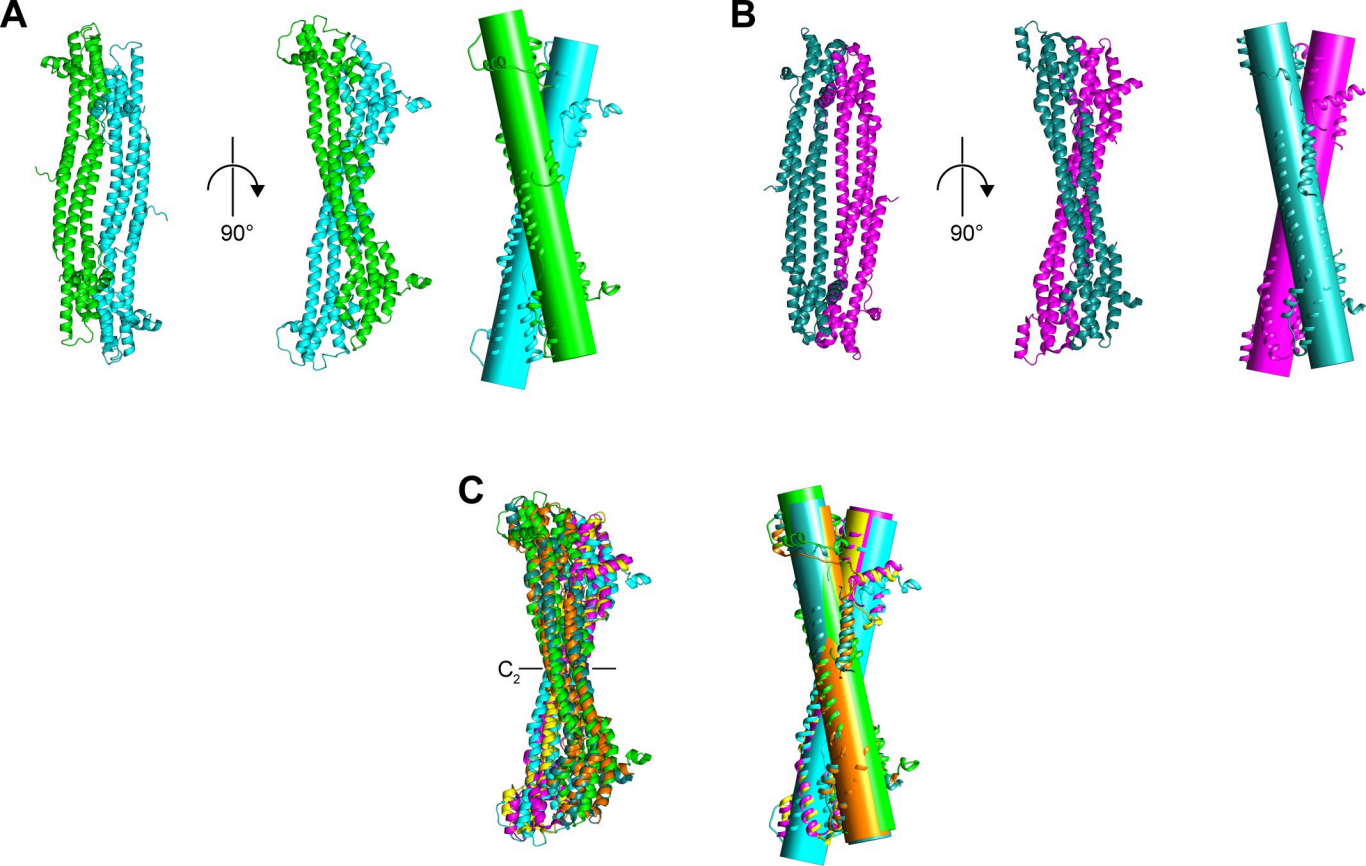
Shared dimerization mode in *rat*IE1_CORE_, *hum*IE1_CORE_ and *rhes*IE1_CORE_. (A) Dimers of IE1_CORE_ proteins are depicted viewing along or perpendicular to the dimerization axis as well as with cylinders placed through all atoms of the respective molecule. (A) *rat*IE1_CORE_, (B) *hum*IE1_CORE_ and (C) superposition of *rat*IE1_CORE_, *hum*IE1_CORE_ and *rhes*IE1_CORE_ (the latter is taken from PDB entry 4WID)_._ The dimeric assembly is characterized by a two-fold rotation axis that interrelates the monomers in the dimer (highlighted in panel C). The cylinder representations show that the two monomer axes of least inertia form an angle of about 23° in the dimers. Panel C shows that this angle is highly similar in *rat*IE1_CORE_ (24.4°), *hum*IE1_CORE_ (22.4°) and *rhes*IE1_CORE_ (21.2°).

All IE1_CORE_ proteins not only display a similar and unique overall fold but also form highly similar dimeric assemblies. In the *rat*IE1_CORE_ and *hum*IE1_CORE_ crystals, the crystallographic asymmetric units contain a single protein chain. However, in both cases, inspection of the crystal packing interactions reveals the presence of tightly interacting dimers (Fig 3A and 3B). In these dimers, the two protomers are related by crystallographic two-fold symmetry axes (Fig 3C) and hence, the dimers display C_2_ point group symmetry similarly to previously described *rhes*IE1_CORE_ [22].

In all IE1_CORE_ structures, the monomers dimerize via an identical interface, and highly similar crossing-angles are adopted between monomers (Fig 3C). The cross-species conserved quaternary arrangement is also evident when comparing the superposition of dimers with the superposition of monomers. When superimposing the various dimers, the calculated rmsd_Cα_ values are only marginally higher than the deviations obtained between monomers in support of a conserved quaternary assembly in IE1_CORE_ proteins (Table 2). Analysis of all IE1_CORE_ structures with program EPPIC suggests that the dimeric assembly corresponds to the biologically active unit of IE1_CORE_ [29]. All remaining protein interfaces observed in the various crystals are classified as mere crystal packing contacts. The sizes of the dimer interfaces are also comparable between IE1_CORE_ proteins ranging from 2240 to 2430 and 2470 Å^2^ in *rat*IE1_CORE_, *hum*IE1_CORE_ and *rhes*IE1_CORE_ (PDB entry 4WIC), respectively. Interestingly, an interface of 2470 Å^2^ is only observed in crystals of *rhes*IE1_CORE_ before induction of a crystallographic phase transition [30]. A dehydration of *rhes*IE1_CORE_ crystals induces a distinct conformational rearrangement in one segment of one protomer of *rhes*IE1_CORE_, and a more extensive dimer interface of about 3070 Å^2^ is formed [22,30].

Taken together, the IE1 proteins appear to form a distinct class of proteins characterized by a shared unique tertiary fold and quaternary assembly. At the same time, the sequence identities observed between these proteins map these to the so-called “twilight zone”, where inference of structural details from sequence alignments only has to be cautioned [25,26].

### The canonical IE1_CORE_ fold is built from conserved regions of left- and right-handed coiled-coils

**Fig 4:**
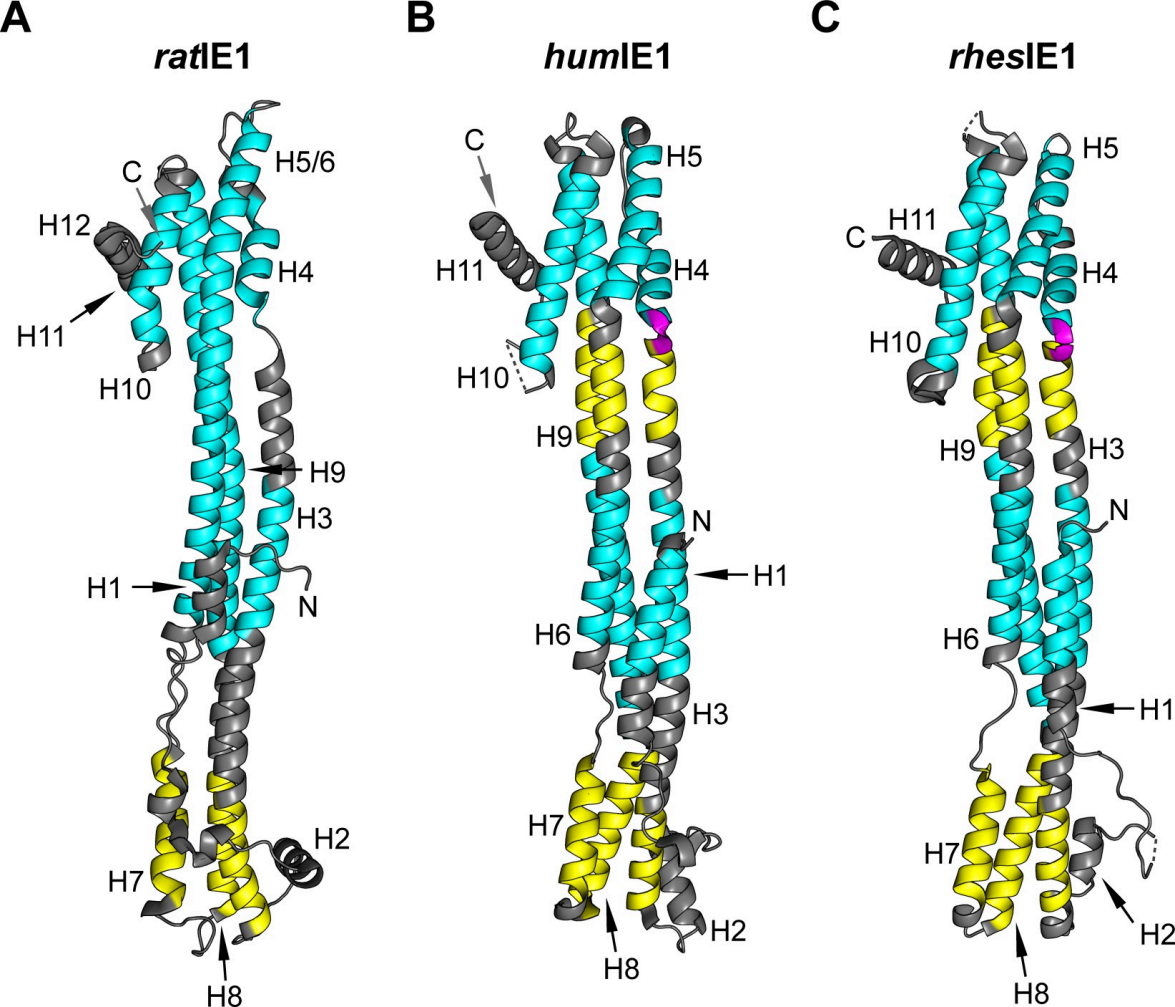
Occurrence and distribution of left- and right-handed coiled-coils in rodent and primate IE1 proteins. Ribbon representation of *rat*IE1_CORE_ (A), *hum*IE1_CORE_ (B) and *rhes*IE1_CORE_ (C) colored according to the handedness of helix-pairings. Yellow: left-handed coiled-coils. Cyan: right-handed coiled-coils. Magenta: three-residue insertion.

The all-α-helical fold of IE1_CORE_ consists of specific left- and right-handed helix pairings that originate from distinct hydrophobic repeat motifs. The N-terminal head region of *rat*IE1_CORE_, *hum*IE1_CORE_ and *rhes*IE1_CORE_ is formed by helices H3, H7 and H8, and these helices form left-handed coiled-coils (Fig 4). The sequences of these helices mainly contain heptad repeats. In these ‘*abcdefg’* repeats, hydrophobic residues are displayed at positions *a* and *d* and give rise to left-handed helix crossings (Figs 4 and S3) [31]. The central stalk and C-terminal head regions exhibit more uncommon, right-handed coiled-coils due to the presence of hendecad (undecad) ‘*abcdefghijk’* repeats with hydrophobic residues at positions *a*, *d* and *h* [31]. However, whereas the stalk and the adjacent C-terminal head region of *rat*IE1_CORE_ are formed by continuous right-handed coiled-coils, this segment is interrupted by a region of left-handed coiled-coils in *rhes*IE1_CORE_ and *hum*IE1_CORE_ (Figs 4 and S3). At the stalk-head transition (H3/H4), *rhes*IE1_CORE_ and *hum*IE1_CORE_ display an insertion of two hydrophilic residues, which point towards the solvent and locally distort the helix geometry to form a sharp kink. In contrast, H3 and H4 of *rat*IE1_CORE_ are separated by a short unstructured linker (Fig 4). The primate CMV proteins further lack one heptad repeat in the middle of H6. Overall, the three IE1_CORE_ structures show similar helix-pairing arrangements. At the same time, specific differences exist in the hydrophobic repeat patterns between the primate and the rodent IE1_CORE_ structures.

These repeat patterns appear more conserved than individual amino acids. Program PROMALS3D was used to generate a structure-based multiple sequence alignment of the three IE1_CORE_ proteins (S3 Fig) [32]. Surprisingly, at nine positions only, amino acid types are conserved across all three proteins. Derivation of pairwise sequence identities from the structure-based alignment reveals that in case of *rhes*IE1_CORE_ and *hum*IE1_CORE_, the observed sequence identity matches that obtained with standard sequence alignment algorithms (22 versus 24%, respectively, Table 2). However, when comparing the structure-derived sequence identity between *rat*IE1_CORE_ and either *hum*IE1_CORE_ or *rhes*IE1_CORE_, sequence identities as low as 9% are obtained for both comparisons. These are considerably lower than the 22% sequence identities obtained with standard sequence alignment algorithms.

Knowledge of the distribution of hydrophobic repeat motifs could help to more reliably model additional IE1-homologous proteins since these distributions are responsible for the topological arrangement of the α-helices in IE1_CORE_. To test this, the sequence of the structurally uncharacterized mouse CMV IE1 (*mur*IE1) protein was manually incorporated into the structure-based sequence alignment of *rhes*IE1, *hum*IE1 and *rat*IE1 (S3 Fig). The alignment shows that the regions can be readily identified and that these show high similarity to those of the crystallized IE1 proteins. We propose that these conserved repeat patterns can be used to improve the reliability of sequence alignments and the correctness of homology models, in particular in cases, where sequence identities fall within the “twilight zone”.

### The mechanism of PML-NB disruption is conserved among primate and rodent cytomegaloviruses

**Fig 5:**
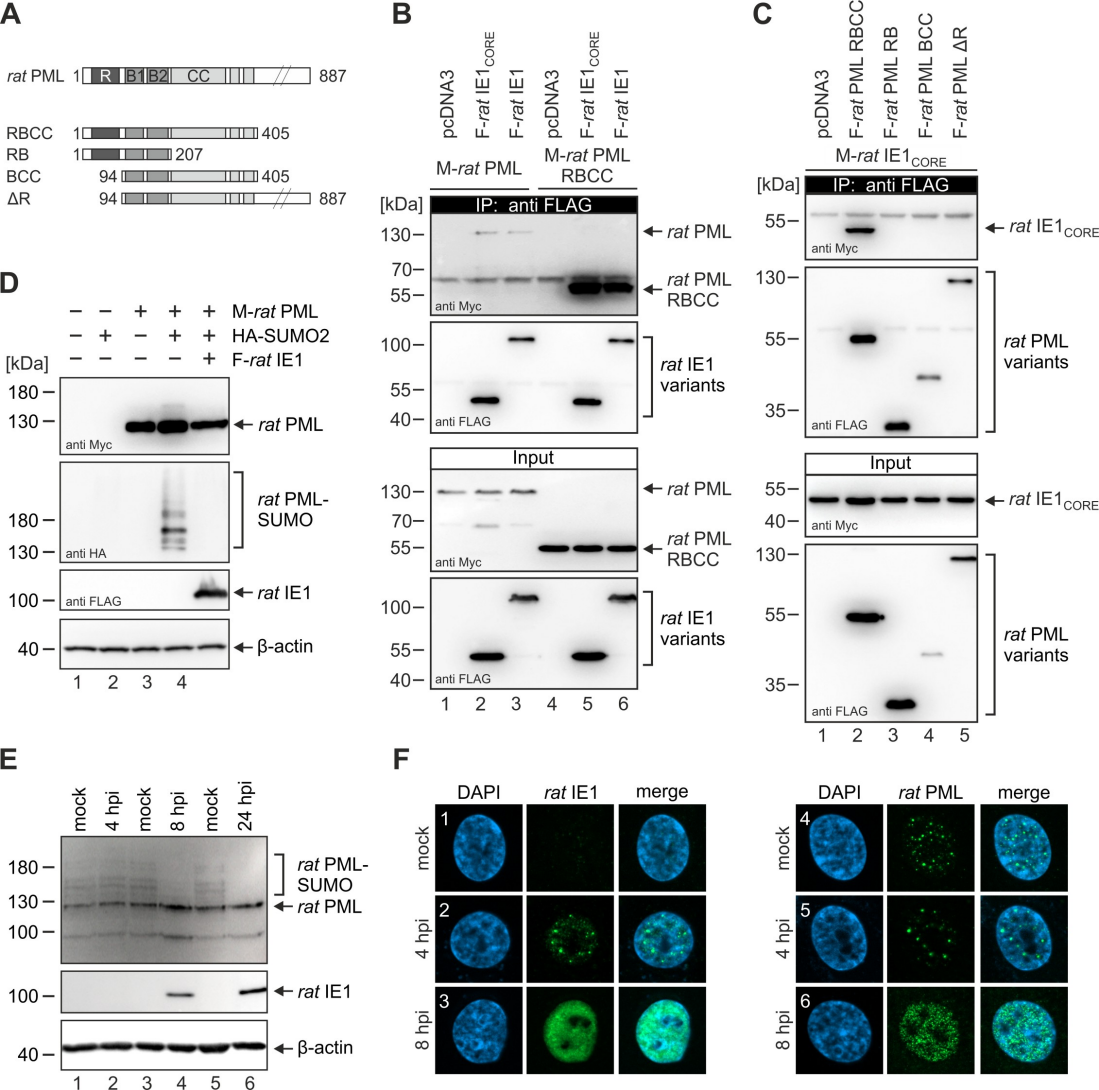
Interaction of *rat*IE1 with *rat*PML followed by *rat*PML deSUMOylation and dispersion. (A) Schematic overview of full-length *rat*PML and deletion mutants. (B) Efficient interaction of *rat*IE1_CORE_ with *rat*PML in co-immunoprecipitation analysis. HEK293T cells were co-transfected with expression plasmids encoding FLAG-tagged *rat*IE1 or *ra*tIE1core (residues 1–392) and Myc-tagged *rat*PML variants. After cell lysis, immunoprecipitation was performed with an anti-FLAG antibody. Co-precipitated *rat*PML proteins (IP), precipitated *rat*IE1 proteins, and proteins within the cell lysate (input) were analyzed by Western blotting as indicated. (C) Binding of *rat*IE1_CORE_ to *rat*PML requires both the coiled-coil and the RING domain. HEK293T cells were co-transfected with expression plasmids encoding FLAG-tagged *rat*PML variants and Myc-tagged *rat*IE1_CORE_ (residues 1–382) as indicated. Upper two panels: Western blot detection of *rat*IE1 and *rat*PML after immunoprecipitation using an anti-FLAG antibody. Lower two panels: detection of *rat*IE1 and *rat*PML in cell lysates before precipitation (input). (D) Inhibition of *rat*PML SUMOylation by *rat*IE1 expression. HEK293T cells were transfected with expression plasmids encoding Myc-*rat*PML, HA-SUMO2 and FLAG-*rat*IE1 as indicated. After cell harvest, *rat*PML and SUMOylated *rat*PML were visualized by Western blotting using anti-Myc and anti-HA antibodies, respectively. Expression of IE1 was analyzed with an anti-FLAG antibody and β-actin was included as internal control. (E) Impact of RCMV infection on *rat*PML SUMOylation. Rat embryonic fibroblast (REF) cells were infected with RCMV at an MOI of 1.5 or mock infected, and were harvested at indicated times for Western Blot analysis of *rat*PML (upper panel), *rat*IE1 (middle panel), and β-actin (lower panel) as loading control. (F) Impact of RCMV infection on *rat*PML-NB integrity. REF cells were infected with RCMV at an MOI of 0.7 or mock infected, and were harvested at indicated times for immunofluorescence analysis of *rat*IE1 (left panel) or *rat*PML (right panel). Cell nuclei were stained with DAPI. F, FLAG; M, Myc; R, RING domain; B, B-boxes; CC, coiled-coil domain.

Due to the structural conservation of the IE1 core domain, the question arose whether all IE1 homologs use the same molecular mechanism to disrupt the antiviral PML-NBs. To address this issue, several rat PML (*rat*PML) deletion mutants were generated and analyzed for an interaction with *rat*IE1 in HEK293T cells (Fig 5A). Co-immunoprecipitation experiments revealed that *rat*IE1 binds full-length *rat*PML and, even more efficiently, the truncated *rat*PML RBCC protein (Fig 5B, lane 3 and 6). A construct encoding an N-terminally extended *rat*IE1_CORE_ protein (*rat*IE1 1–392) was sufficient for this interaction (Fig 5B, lane 2 and 5), which is in accordance with our previous data on human PML (*hum*PML) and *hum*IE1 [22]. Please note that for all cell-based assays, this N-terminally extended IE1_CORE_ variant was used since the N-terminus has been proposed to harbor the NLS signal [33]. Deletion of the coiled-coil domain from the *rat*PML RBCC protein (*rat*PML RB) abolished the interaction with *rat*IE1_CORE_ suggesting that *rat*PML-NBs are targeted through coiled-coil interactions (Fig 5C, lane 3). Proper folding of the *rat*PML RB fragment was confirmed by CD spectroscopy, which also revealed a shared secondary structure composition with the corresponding *hum*PML RB construct (S4 Fig). Moreover, we found that *rat*PML constructs lacking the RING domain (*rat*PML BCC and *rat*PML ΔR) are also not able to bind *rat*IE1_CORE_ (Fig 5C, lane 4 and 5). However, comparatively low expression levels of such constructs in lysate and precipitation samples hint to a possible requirement of the RING domain for proper folding and solubility of *rat*PML.

Next, we examined whether *rat*IE1 induces a loss of *rat*PML SUMOylation and disruption of *rat*PML foci. Transfection experiments using HEK293T cells showed that *rat*IE1 is sufficient to induce a loss of SUMOylated *rat*PML (Fig 5D). To verify this result in the context of infection, rat embryonic fibroblast (REF) cells were either not infected (mock) or infected with RCMV and were analyzed for the SUMOylation state and localization of *rat*PML at immediate-early times. While SUMOylated forms of *rat*PML were still detectable at 4 hours post-infection (hpi) and showed comparable levels as in non-infected cells, we observed a clear loss of *rat*PML SUMOylation beginning at 8 hpi (Fig 5E). In parallel with the depletion of SUMOylated *rat*PML, the intracellular localization of *rat*PML and *rat*IE1 changed from a dot-like to a nuclear diffuse staining pattern (Fig 5F). Since these data match previous findings on *rhes*IE1 and *mur*IE1, which also abrogate PML SUMOylation and induce a dispersion of PML, we conclude that the molecular mechanism underlying PML-NB disruption is conserved across cytomegalovirus species and relies on the unusual fold of the IE1 core domain [22,34].

### PML-NBs are not disrupted during cross-species infection

**Fig 6:**
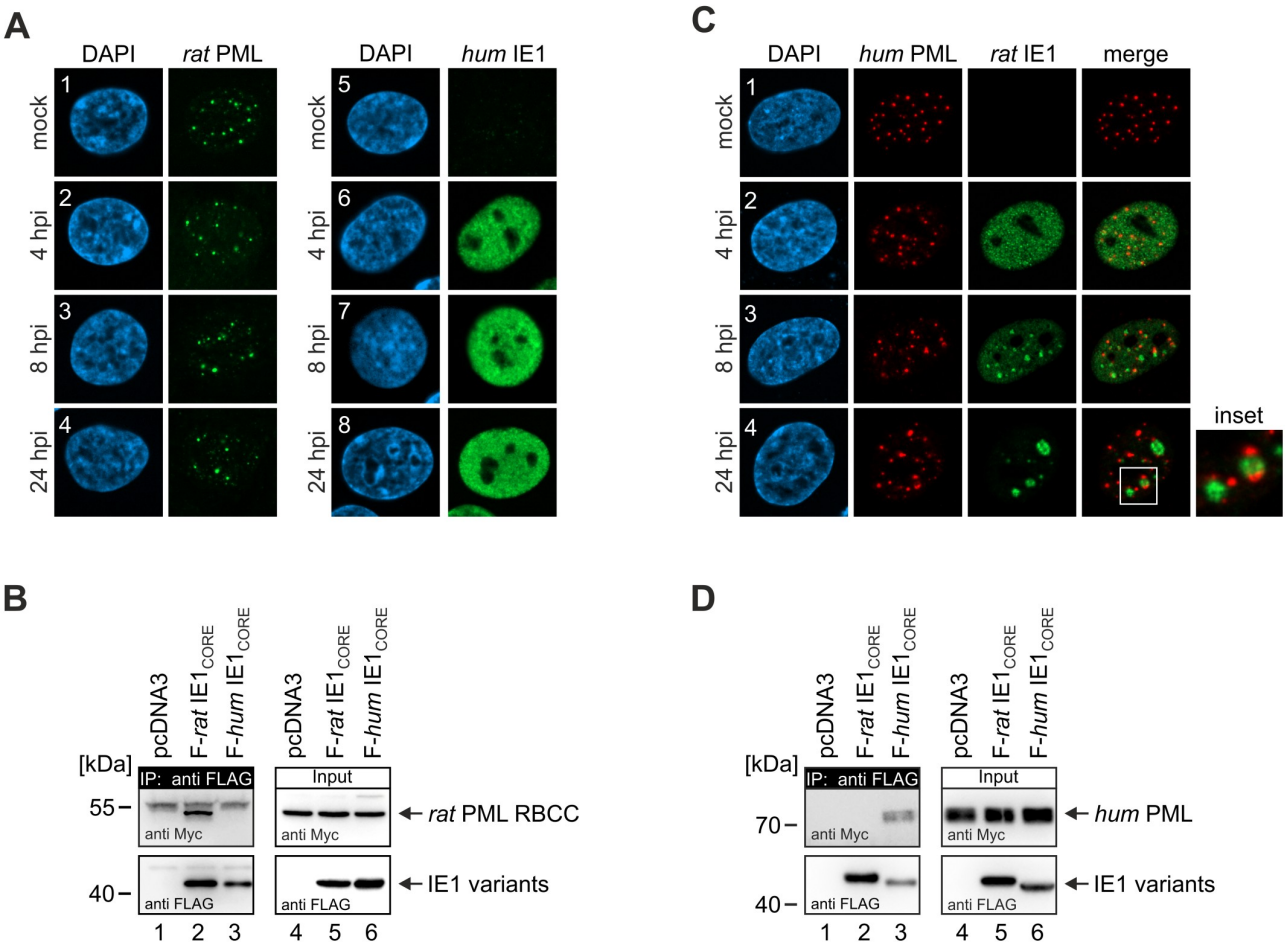
Species-specific disruption of PML-NBs during CMV infection. (A) Analysis of PML-NB integrity in rat fibroblasts after HCMV infection. REF cells were infected with HCMV strain AD169 (MOI = 0.5) or mock infected. Cells were harvested at indicated times after infection to analyze the subcellular localization of *rat*PML (left panel) and *hum*IE1 (right panel). Cell nuclei were stained with DAPI. (B) Species-specific binding of IE1 proteins to *rat*PML in co-immunoprecipitation analysis. HEK293T cells were co-transfected with expression plasmids coding for the TRIM motif of *rat*PML fused to a myc-tag (*rat*PML RBCC) and either FLAG-*rat*IE1_CORE_ (residues 1–392), FLAG-*hum*IE1_CORE_ (residues 1–382) or an empty plasmid (pcDNA3). Afterwards, immunoprecipitation was performed with an anti-FLAG antibody. Left panels: Western blot detection of precipitated IE1 proteins and co-precipitated *rat*PML RBCC (IP). Right panels: detection of IE1 proteins and *rat*PML RBCC in cell lysates before precipitation (input). (C) Analysis of PML-NB integrity in human fibroblasts after RCMV infection. HFF cells were infected with RCMV-E (MOI = 0.5) or mock infected. Cells were fixed at indicated times for immunofluorescence analysis of *hum*PML and *rat*IE1. Cell nuclei were visualized by DAPI staining. (D) Species-specific binding of IE1 proteins to *hum*PML in co-immunoprecipitation analysis. HEK293T cells were co-transfected with expression plasmids encoding myc-tagged *hum*PML and either FLAG-*rat*IE1_CORE_ (residues 1–392), FLAG-*hum*IE1_CORE_ (residues 1–382) or an empty plasmid (pcDNA3). After immunoprecipitation of IE1 with an anti-FLAG antibody, co-precipitated *hum*PML (left panels) as well as proteins in the lysate before precipitation (right panels) were detected by Western blotting.

Due to the structural similarity of primate and rat CMV IE1, we next investigated whether IE1 proteins can counteract the PML-based defense during cross-species infection. As shown in Fig 6A, we found that HCMV is capable of entering REF cells and initiate *hum*IE1 expression. However, *hum*IE1 did not localize to nuclear foci, but was distributed throughout the nucleus and did not affect the integrity of *rat*PML-NBs. In line with this observation, no interaction of *hum*IE1_CORE_ with *rat*PML RBCC was detected in co-immunoprecipitation analysis (Fig 6B, lane 3), suggesting that *hum*IE1 is neither able to bind nor to disrupt PML-NBs in rat cells. In a *vice versa* experiment, we infected primary human fibroblast (HFF) cells with RCMV. We observed no colocalization of *rat*IE1 with *hum*PML at 4 h after RCMV infection, suggesting that it does not target PML-NBs in human cells (Fig 6C). At later stages, however, *rat*IE1 was recruited to large, nuclear domains resembling viral pre-replication compartments. Since PML-NBs were found adjacent to but not colocalizing with these structures (Fig 6C, panel 4) and since no interaction of *rat*IE1_CORE_ with *hum*PML could be detected (Fig 6D, lane 2), it can be assumed that not *hum*PML but another cellular or viral protein is responsible for recruiting *rat*IE1 into nuclear domains. Taken together, these data suggest that PML-NBs are not disrupted by IE1 upon cross species infection and point to a contribution of the PML-based intrinsic defense to the species barrier.

### IE1 induces PML-NB dispersal in a species-specific manner

**Fig 7:**
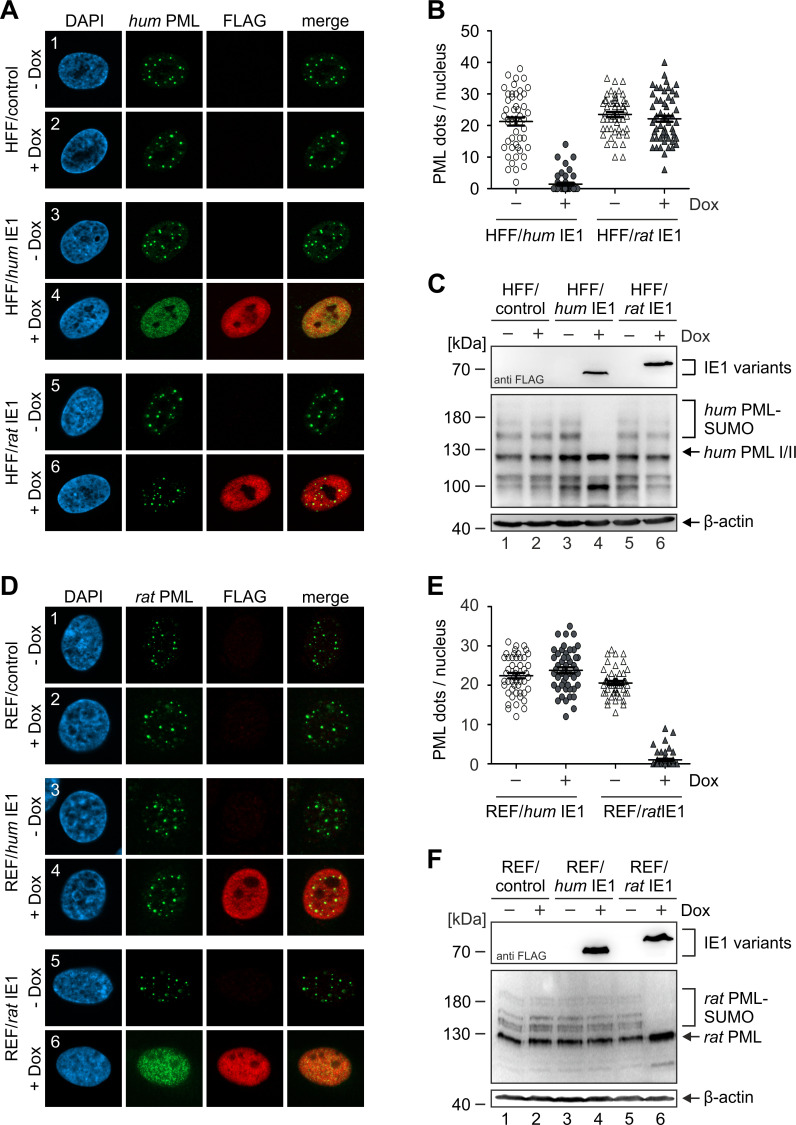
Species-specific disruption of PML-NBs in cells stably expressing IE1. (A, B) Effect of humIE1 and ratIE1 on the integrity of PML foci in human fibroblasts. Human fibroblasts with doxycycline-inducible expression of FLAG-tagged humIE1 (HFF/humIE1), FLAG-tagged ratIE1 (HFF/ratIE1) or control cells (HFF/control) were either left untreated (- Dox) or were treated with doxycycline (+ Dox) for 24 h. The cells were fixed for immunofluorescence staining of endogenous humPML and of IE1 proteins using an anti-FLAG antibody (A), followed by quantitation of humPML foci numbers in 50 cell nuclei per sample (B). (C) Impact of humIE1 and ratIE1 on the SUMOylation state of humPML. HFF/humIE1, HFF/ratIE1 or control cells were either left untreated (- Dox) or were treated with doxycycline (+ Dox). 24 h later, cells were harvested for Western Blot detection of IE1 proteins using an anti-FLAG antibody (upper panel), humPML (middle panel), and β-actin as loading control (lower panel). (D, E) Effect of humIE1 and ratIE1 on the integrity of PML foci in rat fibroblasts. Rat fibroblasts with doxycycline-inducible expression of FLAG-tagged humIE1 (REF/humIE1), FLAG-tagged rIE1 (REF/ratIE1) or control cells (REF/control) were either mock treated (- Dox) or were treated with doxycycline (+ Dox) for 24 h. The cells were fixed for immunofluorescence staining of endogenous ratPML and for IE1 proteins using an anti-FLAG antibody (D), followed by quantitation of ratPML foci numbers in 50 cell nuclei per sample (E). (F) Impact of humIE1 and ratIE1 on the SUMOylation state of ratPML. REF/humIE1, REF/ratIE1 or control REF were either left untreated (- Dox) or were treated with doxycycline (+ Dox). 24 h later, cells were harvested for Western Blot detection of IE1 proteins using an anti-FLAG antibody (upper panel), ratPML (middle panel), and β-actin as loading control (lower panel).

In order to analyze the cross-species activity of IE1 homologs in absence of other viral proteins, we performed a set of experiments using transduced fibroblasts. Lentiviral vectors were utilized to establish human fibroblast (HFF) and rat fibroblast (REF) cells with doxycycline-inducible expression of FLAG-tagged *hum*IE1 or FLAG-tagged *rat*IE1 as well as control cells. Subsequent immunofluorescence analysis of HFF cell populations in absence or presence of doxycycline revealed a clear dispersal of PML foci upon *hum*IE1 expression (Fig 7A, panel 4), whereas *rat*IE1 did neither colocalize with nor disrupt PML-NBs (Fig 7A, panel 6). Quantification of PML foci per cell nucleus corroborated this finding by showing a sharp decline of PML foci in doxycycline-treated HFF/*hum*IE1, while induction of *rat*IE1 expression did not alter the number of PML-NBs (Fig 7B). In accordance, we observed that *hum*IE1, but not *rat*IE1, is able to inhibit the SUMOylation of PML in HFF cells (Fig 7C). Equivalent results were obtained in REF cells since only expression of *rat*IE1 and not *hum*IE1 resulted in dispersal of PML foci (Fig 7D and 7E) and loss of PML SUMOylation (Fig 7F). Overall, our data suggest that the slight structural differences observed in the core domain of primate and rodent IE1 proteins represent evolutionary adaptations to the respective host and result in species-specific targeting of PML-NBs.

### Expression of *hum*IE1 allows RCMV replication in human cells

**Fig 8:**
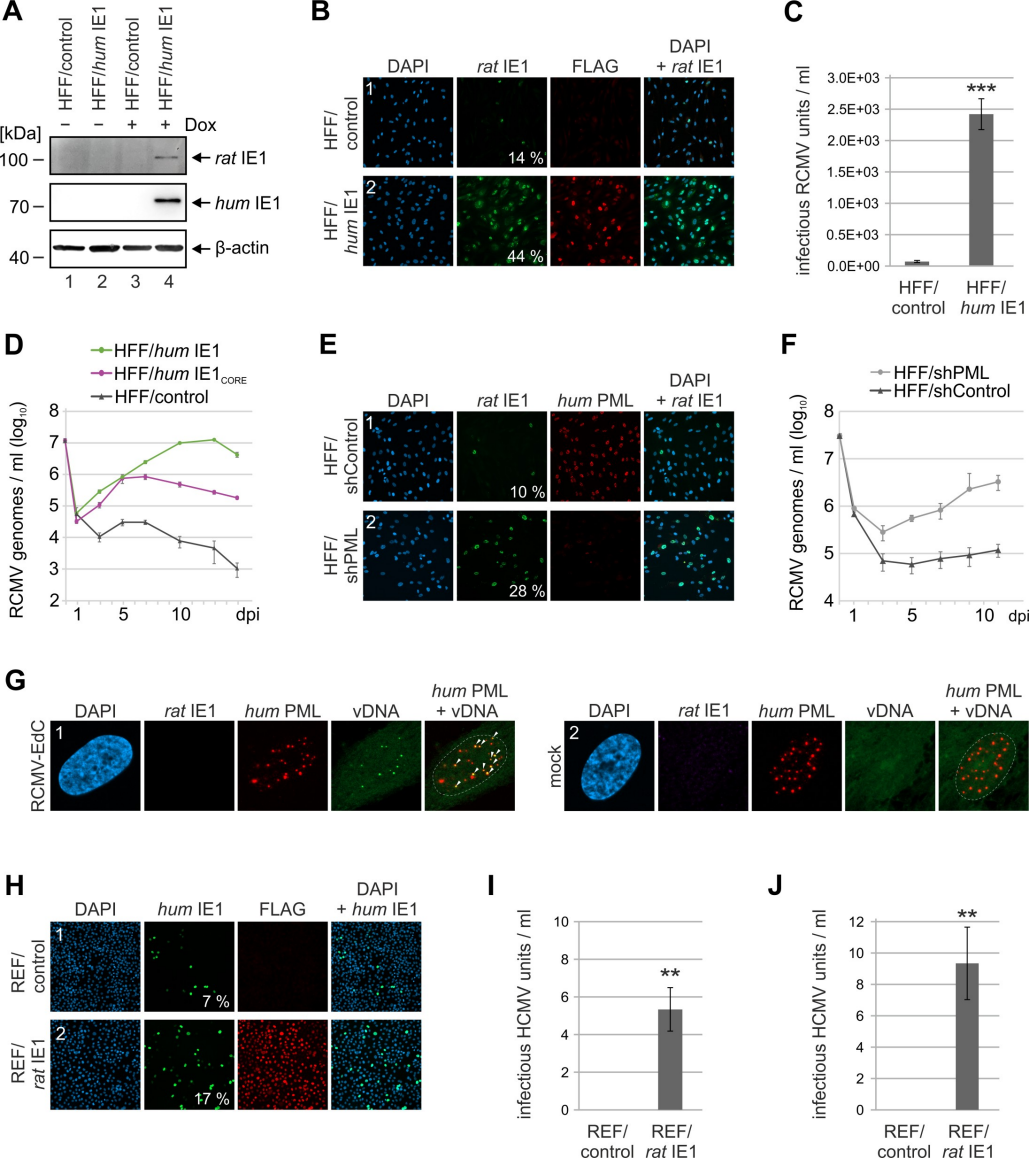
RCMV replication in human fibroblasts expressing *hum*IE1. (A, B) Increased initiation of RCMV gene expression in *hum*IE1-expressing HFF. HFF with doxycycline-inducible expression of FLAG-tagged *hum*IE1 (HFF/*hum*IE1) or control cells (HFF/control) were treated with doxycycline (+ Dox) or mock treated (- Dox) for 24 h and subsequently infected with RCMV-E (MOI = 0.1). At 8 h post-infection (hpi), cells were harvested for Western Blot analysis of *rat*IE1 as well as *hum*IE1 with an anti-FLAG antibody and β-actin as loading control (A) or for immunofluorescence detection of *rat*IE1, *hum*IE1 (FLAG), and cell nuclei by DAPI staining (B). The percentage of *rat* IE1-positive cells was determined from triplicate samples. (C) Release of infectious RCMV particles from *hum*IE1-expressing HFF. HFF/control and HFF/*hum*IE1 were infected with RCMV-E at an MOI of 0.01 after 24 h of doxycycline treatment. Supernatants were harvested at 6 d post infection and titrated on REF cells. Values are derived from triplicate samples and represent mean values ± SD. P-values were calculated using two-tailed Student’s t-test. ***, p ≤ 0.001. (D) Multistep growth curve analysis of RCMV in *hum*IE1-expressing HFF. HFF/control, HFF/*hum*IE1 and HFF/*hum*IE1_CORE_, which express residues 1–382 of *hum*IE1, were treated with doxycycline for 24 h and subsequently infected with RCMV-E at an MOI of 0.01. Supernatants were harvested at indicated times after infection and analyzed for genome equivalents by RCMV gB-specific quantitative real-time PCR. (E) Increased initiation of RCMV gene expression in PML-depleted human fibroblasts. HFF expressing a control shRNA (HFF/shControl) or a shRNA directed against PML (HFF/shPML) were infected with RCMV-E (MOI = 0.1). At 8 hpi, cells were fixed for immunofluorescence detection of *rat*IE1 and *hum*PML. Cell nuclei were visualized by DAPI staining. The percentage of *rat*IE1-positive cells was quantified from triplicate samples. (F) Multistep growth curve analysis of RCMV in PML-knockdown HFF. HFF/shControl and HFF/shPML infected with RCMV-E at an MOI of 0.01. Supernatants were harvested at indicated times after infection and analyzed for genome equivalents by RCMV gB-specific quantitative real-time PCR. (G) Colocalization of RCMV genomes with PML-NBs in human fibroblasts. HFF cells were infected with RCMV-EdC at an MOI of 0.05 or were mock infected. At 8 hpi, cells were fixed for click labeling to visualize RCMV genomes (vDNA) in combination with immunofluorescence detection of *rat*IE1 and *hum*PML. DAPI staining was performed to visualize cell nuclei. Arrows in the merged PML-vDNA image indicate RCMV genomes colocalizing with PML-NBs. Dashed lines indicate the position of the cell nuclei. (H) Increased initiation of HCMV gene expression in *rat*IE1-expressing REF. REF/control and REF/*rat*IE1 were treated with doxycycline for 24 h and subsequently infected with HCMV strain AD169 (MOI = 0.1). At 24 hpi, cells were harvested for immunofluorescence analysis of *hum*IE1, followed by quantification of *hum*IE1-positive cells from triplicate samples. *Rat*IE1 expression was confirmed by staining with an anti-FLAG antibody and cell nuclei were detected with DAPI. (I, J) Release of infectious HCMV particles from *rat*IE1-expressing REF. REF/control and REF/*rat*IE1 were treated with doxycycline for 24h and subsequently infected with HCMV strain AD169 (I) or TB40/E (J) at an MOI of 0.1. Supernatants were harvested at 6 d post infection and directly subjected to titration on HFF cells. Values are derived from triplicate samples and represent mean values ± SD. P-values were calculated using two-tailed Student’s t-test. **, p ≤ 0.01.

Having shown that *rat*IE1 is not able to affect PML-NBs in human cells, we asked whether inactivation of their antiviral activity by providing *hum*IE1 in *trans* results in RCMV particle production. Since PML-NBs are known to block the initiation of lytic replication, we first investigated the effect of *hum*IE1 on RCMV immediate-early gene expression. As shown in Fig 8A (lane 4), low multiplicity infection of human fibroblasts with RCMV yielded detectable levels of *rat*IE1 only when *hum*IE1 expression was induced by doxycycline treatment. This result was confirmed by immunofluorescence analysis, which showed considerably more *rat*IE1-positive cells after infection of doxycycline-induced HFF/*hum*IE1 compared to control HFF, thus suggesting a contribution of PML-NBs to the block of RCMV infection in human cells (Figs 8B and S5). Since previous studies reported that the main block of cytomegalovirus replication in cross-species infection occurs after IE gene expression at the stage of viral DNA replication, we investigated the effect of *hum*IE1 expression on RCMV particle production [35]. For this, supernatant from RCMV-infected HFF/control and HFF/*hum*IE1 was harvested and titrated on REF cells. We found that *hum*IE1 expression indeed stimulates the release of infectious RCMV virions from human fibroblasts suggesting that RCMV can cross the species barrier with help of the HCMV IE1 protein (Fig 8C). In accordance, multistep growth curve analysis showed that RCMV replication occurs in *hum*IE1-expressing HFF, but not in control HFF (Fig 8D). Expression of *hum*IE1_CORE_, which contains the PML binding region but lacks STAT and histone binding sites, also promoted RCMV replication in HFF (HFF/*hum*IE1_CORE_), albeit to lower maximum titers (Fig 8D). Analogous experiments using human fibroblasts depleted for PML (HFF/shPML) as well as control cells (HFF/shControl) likewise revealed an enhanced initiation of RCMV gene expression (Figs 8E and S5) as well as RCMV replication (Fig 8F) in absence of PML, thus further substantiating the role of PML-NBs as a barrier for cross-species infection. Since click-labeling of incoming RCMV genomes in HFF cells revealed a clear colocalization of viral genomes with PML-NBs, but no expression of *rat*IE1, we hypothesize that PML-NBs target RCMV genomes to induce an efficient transcriptional repression (Fig 8G). Finally, we investigated whether HCMV can replicate in rat fibroblasts that overexpress *rat*IE1. We observed a significantly increased initiation of HCMV gene expression in REF/*rat*IE1 compared to control cells (Figs 8H and S5). Titration of the supernatants on fresh HFF cells revealed that only few infectious HCMV particles were released from REF/*rat*IE1 cells, irrespective of whether HCMV laboratory strain AD169 (Fig 8I) or HCMV strain TB40/E (Fig 8J) were utilized. However, no infectious HCMV particles could be detected after infection of REF/control cells (Fig 8I and 8J). In summary, these experiments indicate that disruption of PML-NBs by *rat*IE1 increases HCMV IE gene expression in rat fibroblasts but, in contrast to the significant release of RCMV from human fibroblasts expressing *hum*IE1, results only in low-level HCMV replication.

## Discussion

CMVs are known for their distinct species-specificity resulting from long-term co-evolution with their mammalian hosts [36–38]. Restriction factors can act as important drivers of viral adaptation since they serve as barriers for cross-species transmission necessitating the rapid evolution of viral evasive mechanisms during co-speciation [39,40]. While signatures of positive selection are mainly detected utilizing bioinformatics tools for multiple sequence alignments, protein 3D structures have only recently been integrated into the analysis of adaptive evolution [41]. In this study, we report and compare the structures of human, rhesus and rat cytomegalovirus immediate-early 1 (IE1) proteins, which function as antagonists of the intrinsic cellular defense conferred by PML nuclear bodies (PML-NBs). Our data demonstrate that the IE1_CORE_ domains of primate and rodent cytomegaloviruses form a unique protein class and display high structural similarity, strongly suggesting that this distinct fold represents an evolutionary adaptation to efficiently bind and neutralize PML-NBs.

Surprisingly, the high structural similarity of IE1_CORE_ domains is paralleled by an unexpectedly high dissimilarity at the sequence level. Of the three available IE1_CORE_ structures, *hum*IE1_CORE_ and *rhes*IE1_CORE_ exhibit the closest structural similarity and share 22% sequence identity as deduced from a structure-based alignment (Table 2). In contrast, *rat*IE1_CORE_ displays only 9% of structurally identical residues in comparison to the primate proteins. This value differs significantly from the 22% obtained from pure sequence-based alignments (Table 2). Moreover, the 9% approach the value of 6.5% that is obtained when aligning sequences generated by randomly scrambling the amino acid sequence of *rat*IE1. The discrepancy between structure and sequence-derived identities raises the question of whether the sequence has been registered correctly in the experimentally derived structure since sequence registration errors cannot *per se* be ruled out at resolutions lower than 3 Å [42]. In case of *rat*IE1_CORE_, the correct sequence registration has been corroborated by two additional anomalous scattering experiments, thereby rendering the possibility of registration errors highly unlikely. The low structure-derived sequence identity in combination with the pronounced structural differences explain why it was not possible to solve the structure of ratIE1 via molecular replacement and underline the importance of the experimental structure determination for obtaining an accurate ratIE1_CORE_ structure with all amino acids reliably allocated and surface patches correctly assembled.

The experimental structure of *rat*IE1_CORE_ shows that despite rmsd_Cα_ deviations of up to 4.4 Å, the fold of *rat*IE1_CORE_ still remains similar to the primate variants regarding size and overall architecture of the proteins. Thus, we assume that strong positive selection was responsible for the evolution of a distinct IE1 fold in cytomegaloviruses that inactivates the intrinsic cellular restriction instituted by PML-NBs. Furthermore, one may speculate that the unexpected dissimilarity at the sequence level could be due to genetic divergence driven by strong immune selection pressure since IE1 is known to serve as a major target of the cytotoxic T-cell (CTL) response [43]. Although antigenic adaptation to antibody responses is a well-known process, selection pressure imposed by CTL immunity has only recently been recognized as an additional important player shaping long-term viral evolution [44].

Our results show that primate and rodent CMV IE1 proteins not only share a distinct fold but they also use a conserved mechanism to inactivate PML-NBs. In previous studies, we demonstrated that *hum*IE1 directly interacts with *hum*PML via its core domain, which abrogates the *de novo* SUMOylation of PML followed by the disassembly of PML-NBs [19]. Similarly, the core domain of *rat*IE1 binds to *rat*PML and also induces loss of SUMOylation as well as dispersal of PML-NBs in rat cells (Fig 5). Importantly, binding of both *hum*IE1 and *rat*IE1 to the respective PML proteins depends on coiled-coil interactions, and this correlates with a conserved composition of left- and right-handed coiled-coil motifs that defines the helix pairing and hence the topology of the canonical IE1_CORE_ fold (Fig 4). Thus, although the sequence identities among the investigated IE1 proteins from primate and rodent CMV IE1_CORE_ are low, a high degree of similarity is observed within the coiled-coil repeat motifs across host species orders. We therefore propose that this knowledge will help identifying and validating further members of the IE1_CORE_ family, which might share even lower sequence identities.

Despite an overall similar tertiary fold and quaternary assembly, distinct differences in the helix arrangements and protein surface properties can be observed between primate and rodent IE1_CORE_ structures (Fig 2). While h*um*IE1 and *rhes*IE1 exhibit the highest similarity corresponding to functional exchangeability of the respective proteins in context of a CMV infection [22], *rat*IE1 fails to disrupt PML-NBs in human cells. This correlates with a lack of interaction between *rat*IE1_CORE_ and *hum*PML. Likewise, *hum*IE1 does not bind *rat*PML nor disrupt PML-NBs in rat cells. This is in accordance with previous studies demonstrating that PML-NBs in mouse cells are not disrupted by HCMV and MCMV cannot redistribute PML-NB components in human cells [45]. These data show that rodent and human IE1 proteins function in a species-specific manner strongly suggesting that the IE1 proteins of cytomegaloviruses must have co-evolved with their respective binding partner PML from the host organism.

Interestingly, the sequence dissimilarities observed in the IE1 proteins are not matched by those observed in PML proteins since the latter show a very high degree of sequence conservation. While the sequence identity between the coiled-coil domains of *hum*PML and *rhes*PML is 95%, the sequence identity between these primate domains with the equivalent *rat*PML domain is still as high as approx. 70%. The sequence dissimilarities of the IE1 proteins and the sequence similarities of the PML proteins suggest that the IE1 proteins are likely to contribute most to the IE1-PML interaction specificity profile and thereby control the species specificity of CMVs in antagonizing PML-NB-mediated intrinsic immune defense. A recent study has reported that mutation of four adjacent surface residues within helix 5 of *hum*IE1 is able to abrogate its interaction with *hum*PML [46]. While this segment displays a lysine residue in *hum*IE1 (Lys172) and *rhes*IE1 (Lys187), a glycine residue is displayed at this position in *rat*IE1 (Gly187). Clearly, this substitution could contribute to the observed interaction profile. However, a comprehensive mapping of the IE1-PML binding interface has not been achieved yet and will require further structural and functional investigations.

Cellular restriction factors and their antagonization by viral effector proteins can act as decisive factors for cross-species transmission of viruses. This has extensively been studied for antiretroviral restriction factors like APOBEC3 deaminases which need to be degraded by adapted lentiviral vif proteins in order to allow cross-species infection [47]. In contrast, the mechanisms limiting cross-species infection of cytomegaloviruses are less well understood. For instance, a very recent study reported that the replication of rhesus CMV in human cells is considerably enhanced upon duplication of a terminal genomic region which enhances expression of the protein kinase R (PKR) antagonist rTRS1 encoded by rhesus CMV [48]. This indicates that translational inhibition instituted by human PKR serves as a barrier against cross-species infection with rhesus CMV, which can be overcome by adaptive gene amplification [49]. Furthermore, Jurak and Brune published that infection of human cells with MCMV triggers the intrinsic apoptosis pathway thus abrogating productive viral infection [50]. Expression of the HCMV encoded bcl-2 homolog UL37x/vMIA, however, alleviates this block indicating that induction of apoptosis may contribute to the inhibition of cross-species infections of rodent CMVs. Here, we report that PML-NBs also serve as a distinct barrier against cross-species infections of rodent CMVs. While *rat*IE1 was not able to affect PML-NBs in human cells, we found that inactivation of their antiviral activity by providing *hum*IE1 in *trans* not only resulted in RCMV immediate early gene expression but also in the release of significant amounts of viral particles indicating unrestricted productive infection of human cells by RCMV (Fig 8). Since both the expression of *hum*IE1_CORE_ and the shRNA mediated depletion of PML in human fibroblasts were sufficient to elicit productive RCMV replication, we conclude that the PML-NB disrupting activity of IE1 plays a major role to allow for permissive infection (Fig 8D–8F). Of note, a perfect colocalization of viral DNA with PML-NBs after infection of human fibroblasts with RCMV was observed suggesting efficient silencing of RCMV gene expression in the absence of PML-NB antagonization (Fig 8G). HCMV infection of rat fibroblasts expressing *rat*IE1 also resulted in increased initiation of viral gene expression, however, only low release of viral particles was observed indicating an additional barrier for productive HCMV infection in rodent cells (Fig 8H–8J). This is in accordance with previously published results on MCMV demonstrating that knocking-down of PML-NB components significantly increases viral protein production in cross-species infection experiments, however, does not result in productive infection [34,45]. Interestingly, while PML-NB disruption was sufficient for productive RCMV infection of human fibroblasts, MCMV was shown to require additional HCMV gene functions to cross the human species barrier [35]. In summary, while emerging evidence supports the view that cytomegaloviruses have to counteract multiple hurdles to infect the cells of other species, our study provides strong evidence that PML-NBs-based defense contributes as an important barrier against cross-species infections. Furthermore, our data support the concept that long-term co-speciation of cytomegaloviruses has evolved a distinct IE1 fold that has been adapted to maximize the efficiency of PML-NB targeting.

## Material and methods

### Oligonucleotides and expression plasmids

All oligonucleotide primers used in this study were purchased from Biomers GmbH, Eurofins Genomics GmbH or Metabion GmbH and are listed in S4 Table. Expression plasmids encoding HCMV IE1 (*hum*IE1) 14–382 for prokaryotic expression or *hum*IE1 1–382 for eukaryotic expression were generated as described previously [22]. The codon-optimized RCMV IE1 (*rat*IE1) template cDNA (strain RCMV-E, sequence based on Uniprot K7XWE8) was obtained from Biocat GmbH gene synthesis service. Prokaryotic expression plasmids coding for full length *rat*IE1 and the variants 1–392 and 30–392 were generated by PCR amplification of codon-optimized sequences and insertion into pGEX-6P-1 (GE Healthcare), resulting in GST-tagged fusion proteins. Eukaryotic expression plasmids for co-immunoprecipitations were generated via PCR amplification of respective fragments from plasmids containing the *rat*IE1 cDNA (a kind gift of Sebastian Voigt, Berlin, Germany) or *rat*PML cDNA (sequence based on Uniprot F1M589), which was synthesized by Biocat GmbH. The PCR products were inserted into pHM971 (pcDNA3-FLAG) or pHM1580 (pcDNA3-Myc) [51]. The plasmid encoding Myc-tagged human PML, isoform VI, was described previously [52]. Prokaryotic expression plasmids coding for *rat*PML 1–207 and *hum*PML 20–255 in pGEX-6P-1 were generated as above. For *hum*PML 20–234, a stop codon was inserted into the *hum*PML 20–255 construct by means of site-directed mutagenesis. For transduction experiments, the lentiviral vector pInducer20 (a gift from Stephen Elledge; Addgene plasmid # 44012; http://n2t.net/addgene:44012; RRID:Addgene_44012) was modified by site-directed mutagenesis of the *cis*-repression sequence (CRS) within its promoter region as the CRS leads to transcriptional repression during HCMV infection [53,54]. Mutagenesis was performed with primers c-CRS-mut and nc-CRS-mut and resulted in plasmid pInducer20-CRSmut. FLAG-tagged HCMV IE1 and RCMV IE1 sequences were amplified by PCR with primers listed in S4 Table and were inserted into pInducer20-CRSmut by a combined BP/LR Gateway recombination reaction using pDONR221 (Invitrogen) as entry vector. Lentiviral pLVX-shRNA1-based vectors containing a control shRNA or a shRNA directed against PML were generated as described previously (see S4 Table for target sequences) [55].

### Recombinant protein production and purification

All variants of IE1 and PML were recombinantly produced in *E*. *coli* BL21(DE3) cells (Novagen) as GST-tagged fusion proteins. LB or TB media (Carl Roth) were inoculated with transformed *E*. *coli* cells and shaken at 37°C. Media were supplemented with 100 μg/mL of ampicillin as well as 50 μM ZnCl_2_ for the expression of *hum*PML. Overexpression was induced by adding 0.1 mM IPTG and shaking at 20°C over night. Seleno-methionine labeled *rat*IE1 30–392 was produced in auto-inducing PASM-5052 medium as described in the literature [56].

All purification steps were performed at 4–8°C. All chromatography buffers contained 5 mM DTT and either 1 mM EDTA (IE1 variants) or 25 μM ZnCl_2_ (PML variants). Cell pellets were resuspended in PBS buffer and lysed by sonication. Fusion proteins were captured using the aforementioned affinity media. GST-fusion proteins were cleaved with a GST-tagged human rhinovirus 3C protease and purified using a second affinity chromatography step. Proteins were then concentrated using Vivaspin 20 centrifugal concentrators (5 kDa molecular weight cutoff, Sartorius Stedim) and purified using a 26/600 Superdex 200 prep grade column (GE Healthcare) pre-equilibrated in 25 mM TRIS/HCl, 150 mM NaCl, 5 mM DTT, pH 7.4. The samples were eluted with an isocratic gradient of 1.2 column volumes of the same buffer at a flow rate of 34 cm/h (3 mL/min).

For crystallization, surface lysine residues of *rat*IE1 30–392 were chemically methylated. This was performed after the second affinity chromatography step. The buffer was exchanged to 25 mM Na-HEPES, 150 mM NaCl, 5 mM DTT, 1 mM EDTA, pH 7.5 and 20 μL of a 1 M borane dimethylamine complex and 40 μL 1 M formaldehyde were added per mL of protein solution. After two hours of incubation on ice, the addition of borane dimethylamine complex and formaldehyde was repeated. After additional two hours, another 10 μL of dimethylamine borane complex per mL of solution were added and the reaction was incubated over night. The reaction was quenched by adding 125 μL 1 M TRIS/HCl pH 7.5 per mL solution. The methylated protein samples were purified by size exclusion chromatography as described above.

### Limited proteolysis

Full-length *rat*IE1 was incubated at 22°C with 1 mU subtilisin (Sigma-Aldrich) per mg *rat*IE1 in the presence of CaCl_2_. 15 μL samples were taken at timepoints between one and 128 min and immediately mixed with 5 μL 4x SDS PAGE loading buffer and boiled at 95°C for 5 min. A gel band was excised and analyzed by mass spectrometry.

### Circular dichroism spectroscopy

Circular dichroism spectra were recorded between 185 and 260 nm using a Jasco J-815 spectropolarimeter (Jasco, Tokyo, Japan). Protein samples were dialyzed twice against 10 mM KH_2_PO_4_ pH 7.5 and set to a concentration of 5 μM. Measurements were conducted at 20°C using a quartz cuvette with a path length of 0.1 cm, scan speed of 20 nm/min, band width 1 nm, data integration time 1 s and data pitch 0.1 nm. All measurements were accumulated eight times and corrected for the sample buffer. The spectra were normalized at 207 nm as suggested by Raussens and coworkers [57].

### Protein crystallization

Prior to crystallization, all proteins were dialyzed against an at least 500-fold volume of 25 mM TRIS/HCl, 10 mM DTT, 1 mM EDTA, pH 7.4 (set at 20°C) and concentrated to 20 mg/mL. Initial crystallization screening was performed using commercial screens and the sitting drop vapor diffusion method. Diffraction quality crystals were obtained in the hanging drop setup. *Rat*IE1_CORE_ and *hum*IE1_CORE_ crystallized with reservoir solutions of 0.1 M TRIS/HCl pH 8.5, 0.9 M MgCl_2_ x 7 H_2_O at 4°C or 50 mM Na-malonate pH 5.0, 9% (w/v) PEG 3350 at 19°C in conjunction with microseeding, respectively.

### Crystallographic data collection

Data of native and seleno-methionine derivatized *rat*IE1 30–392 crystals were collected at 100 K at beamline 14.2 at the BESSY II synchrotron ([58]; Helmholtz Zentrum Berlin). The protein crystallized in the space group P6_5_22 and the crystal diffracted to 3.4 Å. In addition to a native dataset, two-wavelength MAD data were collected from seleno-methionine derivatized crystals (peak, inflection point). For sequence validation, 6 keV anomalous sulfur data were collected from native crystals at the P13 beamline at DESY ([59]; Deutsches Elektronen-Synchrotron, Hamburg). Native data of *hum*IE1 14–382 crystals were collected at the P13 beamline at DESY ([59]; Deutsches Elektronen-Synchrotron, Hamburg). The protein crystallized in the space group C222_1_ and the crystal diffracted to 3.2 Å. All diffraction data were processed using XDS and scaled and merged with XSCALE and XDSCONV [60].

### Structure determination and refinement

The structure of *rat*IE1 30–392 was solved using two-wavelength MAD data from five merged seleno-methionine peak and one inflection point dataset. Anomalous data to 4 Å were used for data preparation (SHELXC), substructure search (SHELXD) and initial chain tracing (SHELXE) [61] as implemented in the HKL2MAP package [62]. The initial phases allowed for the calculation of an anomalous map from the peak data to 5 Å using FFT [63] from the CCP4 suite [64]. This map was used to place the selenium atoms of 11 out of 12 seleno-methionine residues. Based on the methionine positions, the model was built by hand and refined against the native 3.4 Å data using multiple iterations of phenix.refine [65], phenix.rosetta_refine [66] and manual fitting in COOT [67]. Atomic displacement parameters were refined using two B-factors per amino acid residue and three TLS groups per molecule. For further validation of the built sequence, anomalous maps were calculated using phases from the refined model and merged 6 keV X-ray data from seven crystals to a resolution of 5 Å, as well as the aforementioned seleno-methionine peak data.

The dataset from *hum*IE1 was found to be moderately anisotropic. For ellipsoidal truncation, unmerged data were processed using the STARANISO server [68]. The structure of *hum*IE1 14–382 was solved with MR-Rosetta using *rhes*IE1_CORE_ (PDB: 4WID; [22]) as a search model [23]. A poly-alanine model was placed in the obtained electron density. Sequence and structure alignments with the published rhesus CMV IE1 structure (PDB: 4WID) were used to place the sequence. Refinement was conducted as described above. Atomic displacement parameters were refined using two B-factors per amino acid residue and eight TLS groups per molecule. Data collection and refinement statistics are summarized in Table 1.

### Bioinformatic analyses

Sequence based disorder prediction was performed using IUPred2A using the default setting “IUPred2 long disorder” [69]. The dimer interface areas were calculated using the EPPIC server [29]. Pairwise structure-based sequence identities and RMS deviations were calculated using DALI [70]. For comparison of the dimers, coordinates files for *hum*IE1 and *rat*IE1 were generated from the molecule in the asymmetric unit and a symmetry related molecule using COOT [67]. The multiple structure-based sequence alignment was generated with PROMALS3D [32] using the abovementioned structures. Structure-based sequence identities were determined from the structurally equivalent areas of the alignment. Sequence alignments without structural information were obtained using Clustal Omega [71]. Crystal structure illustrations were generated with PyMol and UCSF Chimera [72,73]. The angles between IE1 monomers were determined with the “Axes/Planes/Centroids” tool implemented in Chimera. Axes were placed through all atoms in each monomer.

### Cells and virus infections

HEK293T cells were cultivated in Dulbecco’s minimal essential medium (DMEM) containing glutamine and supplemented with 10  % fetal calf serum and penicillin-streptomycin (Sigma). Primary human foreskin fibroblasts (HFF), which were prepared from human foreskin tissue, and rat embryonic fibroblasts (REF), which were obtained from Sebastian Voigt (Berlin, Germany) were maintained in Eagle’s minimal essential medium (MEM) supplemented with 7 % fetal calf serum (Sigma), glutamax (Gibco), and penicillin-streptomycin (Sigma). HFF and REF cells were infected with either the HCMV strains AD169 and TB40/E or the RCMV English isolate (RCMV-E, kindly provided by Sebastian Voigt) at specified multiplicities of infection (MOI). Viral titers were determined by IE1 fluorescence. To this end, HFF or REF cells were infected with various dilutions of virus stocks. Cells were incubated for 24 h for titration of AD169 or TB40/E and 8 h for titration of RCMV-E, and were subsequently fixed and stained with a monoclonal antibody against *hum*IE1 or *rat*IE1. The number of IE1-positive cells was determined and used to calculate viral titers.

### Lentiviral transduction and selection of stably transduced cells

For the generation of HFF and REF cells with doxycycline-inducible expression of *hum*IE1 or *rat*IE1, replication-deficient lentiviruses were generated using pInducer20-based expression constructs. For this purpose, HEK293T cells seeded in 10 cm dishes (5 x 10^6^ cells/dish) were transfected with a pInducer20-based vector together with packaging plasmids pLP1, pLP2, and pLP/VSV-G using the Lipofectamine 2000 reagent (Invitrogen). Viral supernatants were harvested 48 h after transfection, clarified through a 0.45-μm filter and stored at -80°C. HFF or REF cells were incubated for 24 h with lentivirus supernatants in the presence of 7.5 μg/mL polybrene (Sigma-Aldrich). Stably transduced, IE1-expressing cell populations were selected by adding 500 μg/mL geneticin (Invivogen) to the cell culture medium containing 7 to 10% tetracycline-free fetal bovine serum (Clontech). IE1 expression was induced by addition of 500 ng/mL doxycycline (Sigma-Aldrich). To generate PML-knockdown and control cells, HFF were transduced with lentiviral supernatants produced from pLVX-shRNA-based expression constructs, followed by selection with 5 μg/mL puromycin (Invivogen).

### Co-immunoprecipitation

HEK293T cells were seeded in 6-well plates (7 x 10^5^ cells/well, 2 wells per sample) and, one day later, were transfected with 2 to 4 μg of plasmid DNA per well using the TurboFect transfection reagent (Thermo Fisher Scientific). 48 h after transfection, the cells were lysed for 25 min at 4°C in 800 μL of CoIP buffer (50 mM Tris-HCl [pH 8.0], 150 mM NaCl, 5 mM EDTA, 0.5% NP-40, 1 mM PMSF, 2 μg/mL of aprotinin, 2 μg/mL of leupeptin, and 2 μg/mL of pepstatin). After centrifugation, aliquots of each sample were taken as input controls and the remaining supernatant was incubated with anti-FLAG antibody M2 (Sigma-Aldrich) coupled to protein-A-sepharose beads for 2 h at 4°C. The sepharose beads were collected by centrifugation and washed four times with 1 mL CoIP buffer. Finally, immunoprecipitated proteins were recovered by boiling in 4x SDS sample buffer and protein complexes were analyzed by SDS-PAGE and Western blotting.

### Western blotting

Lysates from transfected or infected cells were prepared in a sodium dodecyl sulfate-polyacrylamide gel electrophoresis (SDS-PAGE) loading buffer by boiling for 10 min at 95°C and sonication for 1 min. Proteins were separated on sodium dodecyl sulfate-containing 8 to 15% polyacrylamide gels and transferred to PVDF membranes (Biorad), followed by chemiluminescence detection using a FUSION FX7 imaging system (Vilber). Following antibodies were used: mAb FLAG M2 (Sigma-Aldrich), mAb Myc 9E10 (1-9E10.2; ATCC), mAb β-actin AC-15 (Sigma-Aldrich), mAb HCMV IE1 p63-27 [74], mAb RCMV IE1 (kindly provided by Sebastian Voigt), mAb PML 5E10 (kindly provided by Roel van Driel) was used to detect *rat*PML, and pAb PML A301-167A and A301-168A (Bethyl Laboratories) were used in combination to detect human PML.

### Indirect immunofluorescence

HFF or REF cells grown on coverslips in six-well plates (3 x 10^5^ cells/well) or 12-well plates (1.2 x 10^5^ cells/well) were washed twice with PBS at specified times after virus infection or after doxycycline treatment. Cells were fixed with a 4% paraformaldehyde solution for 10 min at room temperature (RT) and washed twice, before permeabilization was achieved by incubation with 0.1% Triton X-100 in PBS for 10 min on ice. Cells were washed again with PBS over a time period of 5 min and incubated with the appropriate primary antibody diluted in 1% FCS in PBS for 30 min at 37°C. Excessive antibodies were removed by washing three times with PBS, followed by incubation with the corresponding fluorescence-coupled secondary antibody diluted in 1% FCS in PBS for 30 min at 37°C. The cells were mounted with DAPI-containing Vectashield mounting medium (Vector Laboratories) and analyzed using a Zeiss Axio Observer Z1 with an Apotome.2. The images were processed and exported with the ZEN 2 software and assembled using CorelDraw 2018. For quantification of PML foci, Z-series images of 50 cell nuclei per sample were taken and the number of PML dots was assessed in maximum intensity projection images (0.3 μm distance). Following antibodies were used for immunofluorescence detection: mAb FLAG M2 (Sigma-Aldrich), mAb HCMV IE1 p63-27 [74], mAb RCMV IE1 (kindly provided by Sebastian Voigt), mAb PML 5E10 (kindly provided by Roel van Driel) to detect rat PML, and pAb PML A301-167A (Bethyl Laboratories) to detect human PML.

### RCMV DNA labeling with ethynyl-modified nucleosides and detection by click chemistry

In order to produce labeled RCMV stocks, RCMV-E was grown in REF cells in the presence of 5μM EdC. EdC-containing medium was replaced every 24 h until a strong cytopathic effect was observed. Supernatants from infected cells were clarified by centrifugation at 2000 rpm for 15 min and then pelleted by ultra-centrifugation at 17000 rpm for 3 h at 4°C. Pellets were rinsed with medium, before they were resuspended and passed through a 20 gauge syringe needle for several times. In order to visualize viral DNA in combination with antibody staining, HFF infected with EdC -labeled RCMV were fixed with 4% PFA for 10 min and quenched with 50 mM ammonium chloride and 50 mM glycine in PBS for 5 min at RT. Cells were washed twice with PBS, permeabilized with 0.1% TritonX100 in PBS for 15 min at 4°C, and stained with antibodies as described above. After washing cells twice with PBS, copper-catalyzed click reaction was performed by incubating the cells for 90 min at RT with freshly prepared labeling solution containing 10 μM Alexa Fluor 488 Azide (Invitrogen), 10 mM sodium ascorbate, 1 mM copper (II) sulfate, 10 mM aminoguanidine, and 1 mM THPTA in PBS. Cells were washed twice with PBS for 5 min, before coverslips were mounted on microscope slides using Vectashield Antifade Mounting Medium with DAPI (Vector laboratories, Maravai LifeSciences, San Diego, CA, USA) and sealed with nail polish.

### Multistep growth curve analysis

HFF cells were seeded in triplicates or quadruplicates into 12-well dishes at a density of 1.2 × 10^5^ cells/well and were treated with doxycycline for one day, before they were infected with RCMV at an MOI of 0.01. Supernatants from infected cells were harvested at indicated days after inoculation and cells were provided with fresh medium containing doxycycline. After proteinase K treatment, all samples were analyzed for the amount of genome copy numbers by quantitative real-time PCR using an Agilent AriaMx Real-time PCR System together with the corresponding software Agilent Aria 1.5 (Agilent Technologies, Inc, Santa Clara, CA, USA). For quantification of the RCMV DNA load, a sequence region within the gB gene locus was amplified using primers 5’RCMV-and 3’RCMV-gB along with the hydrolysis probe RCMV-gB FAM/TAMRA (S4 Table) [75]. Real-time PCR was conducted in 96-well plates in 20 μL reactions containing 5 μL sample or standard DNA together with 10 μL 2x SsoAdvanced Universal Probes Supermix (Biorad), 1 μL of each primer (5 μM stock solution), 0.3 μL of probe (10 μM stock solution), and 2.7 μL of H_2_O. For determination of reference C_T_ values (cycle threshold), serial dilutions of the respective standards (10^8^−10^2^ DNA molecules of RCMV gB) were examined by PCR reactions in parallel. The thermal cycling conditions consisted of an initial step of 3 min at 95°C followed by 40 amplification cycles (10 s at 95°C, 30 s 60°C). Viral genome copy numbers were subsequently calculated using the sample-specific C_T_ value set into relation to the standard serial dilutions.

## Supporting information

S1 Fig Sequence coverage of the 45 kDa fragment of ratIE1 obtained upon limited proteolysis of full-length ratIE1. The fragment was analyzed with LC-mass spectrometry post trypsin digestion.
(TIF)

S2 Fig Backbone comparison of r*at*IE1_CORE_, *hum*IE1_CORE_ and *rhes*IE1_CORE_. Backbone representation of *rat*IE1 (green), *hum*IE1 (teal) and *rhes*IE1 (orange) after superposition with DALI. For a better comparison, the superimposed structures were split at the residues marked with a black circle (*rat*IE1 and *rhes*IE1: residue 215, *hum*IE1: residue 200). (A) *Rat*IE1 residues 33–215 and *hum*IE1 residues 25–200. (B) *rat*IE1 residues 215–392 and *hum*IE1 residues 200–382. (C) *Rhes*IE1 residues 41–215 and *hum*IE1 residues 25–200. (D) *rhes*IE1 residues 215–393 and *hum*IE1 residues 200–382.
(TIF)

S3 Fig Structure-based sequence alignment of *rhes*IE1, *hum*IE1 and *rat*IE1. Structure-based sequence alignment calculated with PROMALS3D [32]. The sequences of *rhes*IE1, *hum*IE1 and *rat*IE1 were aligned according to the experimental structures. Helix designations were taken from *rhes*IE1 structure (PDB: 4WID:B). Residues identical in all three structures are marked by an asterisk (*). The handedness of coiled-coils in the structures of *rhes*IE1, *hum*IE1 and *rat*IE1 is marked in yellow (left-handed) or cyan (right-handed). The hydrophilic residues of the three-residue insertions are marked in magenta. Residues occupying the *a*, *d* or *h* positions of heptad or hendecad repeats are shown in boldface. Regions without possible repeats are printed in lower case. The sequence of *mur*IE1 was manually fitted to the aligned sequences. Putative residues involved in heptad or hendecad repeats are indicated as described above.
(TIF)

S4 Fig CD spectroscopy analysis of *hum*PML and *rat*PML variants containing the RB domains. CD spectra of *hum*PML 20–234 (*hum*PML RB) and *rat*PML 1–207 (*rat*PML RB). The spectra were normalized at 207 nm as suggested by Raussens and coworkers [57]. The spectra suggest that the *hum*PML and *rat*PML RB segments share a highly similar secondary structure composition and that both protein variants are properly folded.
(TIF)

S5 Fig Initiation of lytic CMV infection upon cross-species infection. (A, B) *Hum*IE1 expression in HFF enhances RCMV IE gene expression. Control HFF or *hum*IE1-expressing HFF were treated with doxycycline for 24h, followed by RCMV-E infection at an MOI of 0.1 (A) or with a low input of < 500 IE units per well (B). 8 hpi, cells subjected to immunofluorescence staining of *rat*IE1 in order to determine the initiation of lytic gene expression. (C, D) PML depletion from HFF enhances RCMV IE gene expression. Control HFF or PML-knockdown HFF were infected with RCMV-E at an MOI of 0.1 (C) or with a low input of < 500 IE units per well (D). 8 hpi, cells subjected to immunofluorescence staining of *rat*IE1 in order to determine the initiation of lytic gene expression. (E, F) *Rat*IE1 expression in REF enhances HCMV IE gene expression. Control REF or *rat*IE1-expressing REF were infected with HCMV strain AD169 at an MOI of 0.1 (E) or with a low input of 250 IE units per well (F). 24 hpi, cells were subjected to immunofluorescence staining of *hum*IE1 in order to determine the initiation of lytic gene expression. All values are derived from triplicate samples and represent mean values ± SD. P-values were calculated using two-tailed Student’s t-test. **, p ≤ 0.01; ***, p ≤ 0.001.
(TIF)

S1 Table Peak assignment in an anomalous difference map calculated with the selenomethionine peak data. (DOCX)

S2 Table Peak assignment in an anomalous difference map calculated with a long-wavelength data set (2.0 Å, 6 keV). (DOCX)

S3 Table *Hum*IE1_CORE_ and *rat*IE1_CORE_ homologous protein structures as identified using the Dali webserver. (DOCX)

S4 Table Oligonucleotides. (DOCX)
