# Supplementary figures and images for: Cytomegalovirus immediate-early 1 proteins form a structurally distinct protein class with adaptations determining cross-species barriers
Source: PLoS Pathog. 2021 Aug 9;17(8):e1009863. doi: 10.1371/journal.ppat.1009863 (PMC8376021; doi:10.1371/journal.ppat.1009863)

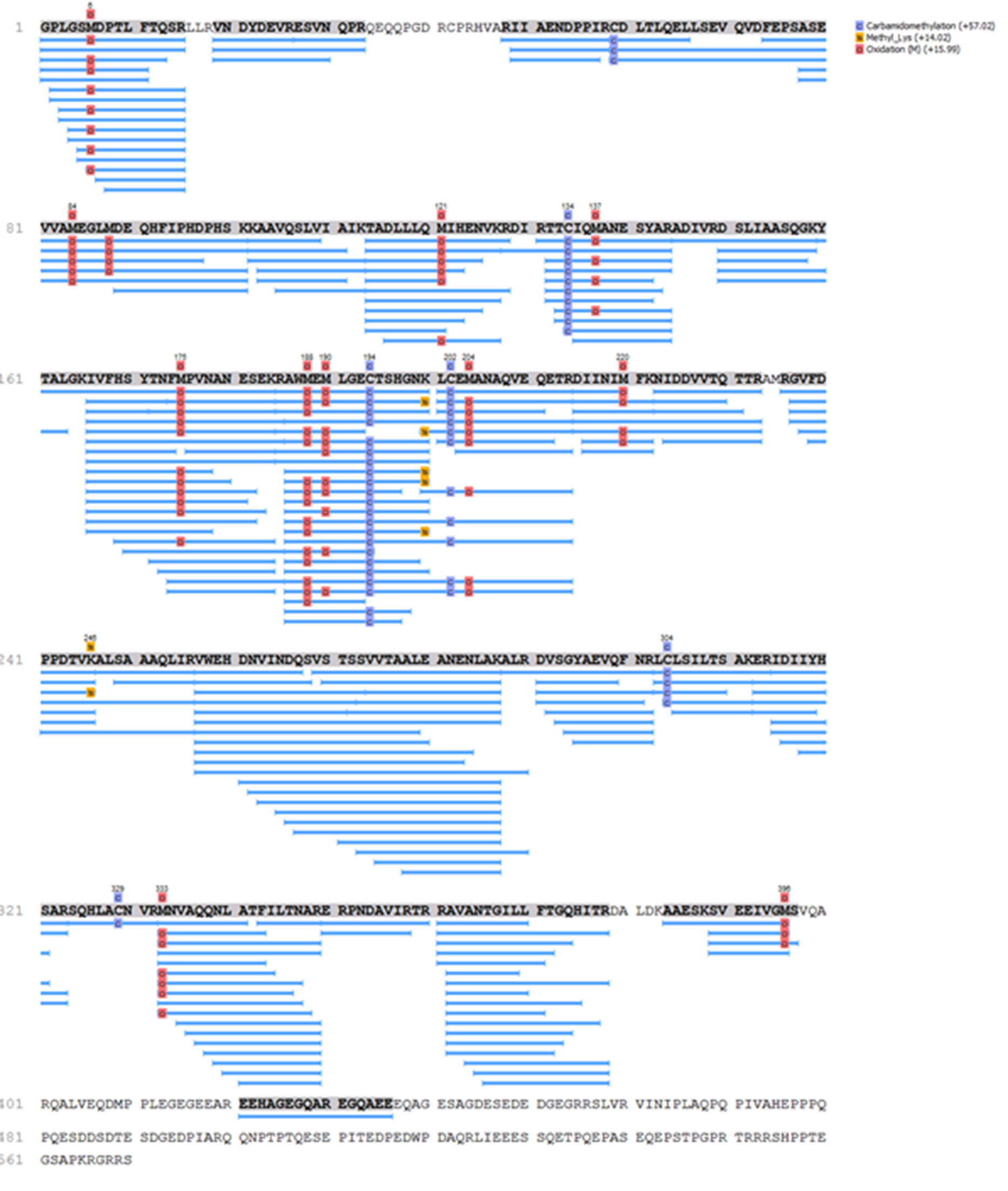

Supplement: S1 Fig — The fragment was analyzed with LC-mass spectrometry post trypsin digestion. (TIF) [file ppat.1009863.s001.tif]

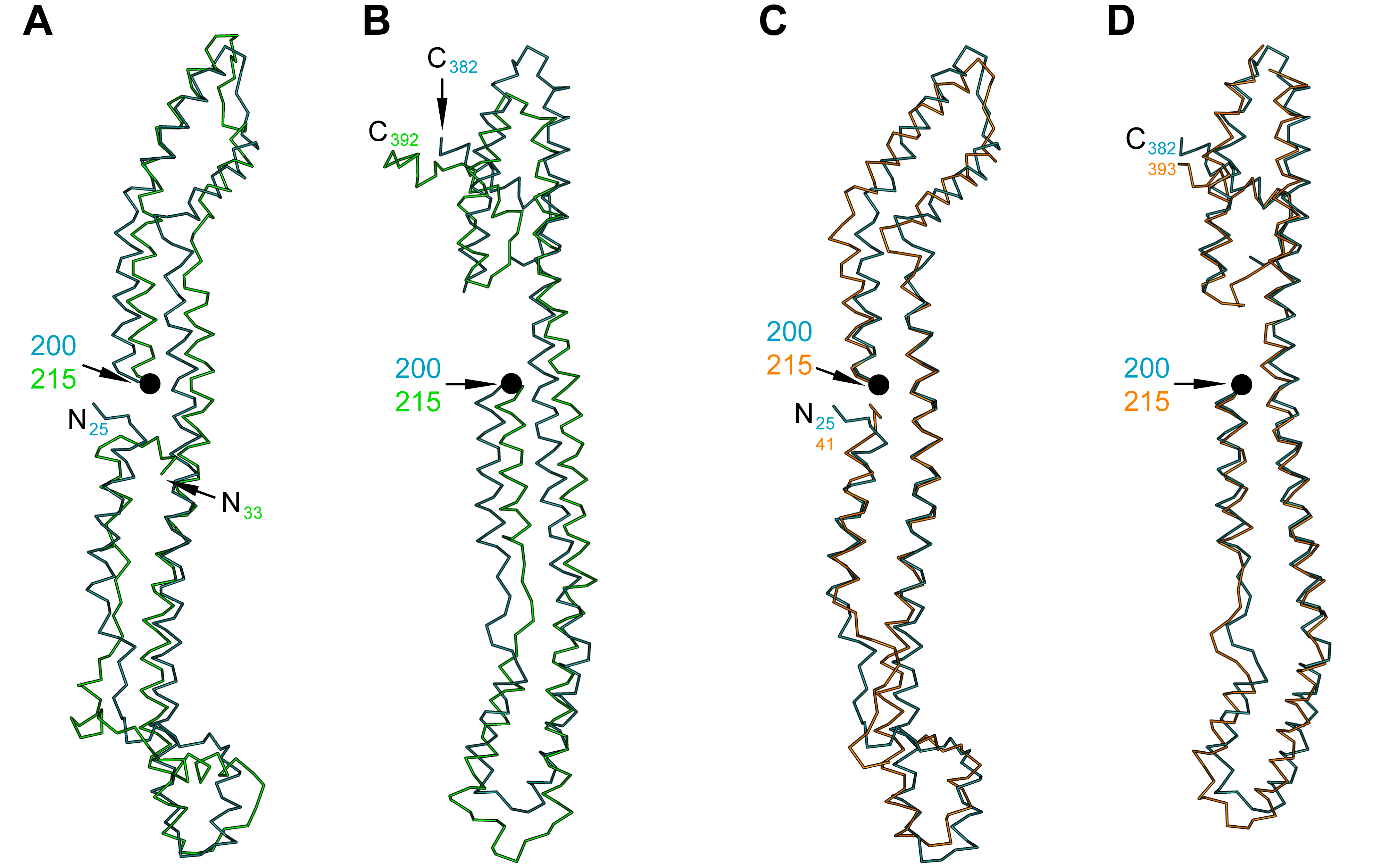

Supplement: S2 Fig — Backbone representation of ratIE1 (green), humIE1 (teal) and rhesIE1 (orange) after superposition with DALI. For a better comparison, the superimposed structures were split at the residues marked with a black circle (ratIE1 and rhesIE1: residue 215, humIE1: residue 200). (A) RatIE1 residues 33–215 and humIE1 residues 25–200. (B) ratIE1 residues 215–392 and humIE1 residues 200–382. (C) RhesIE1 residues 41–215 and humIE1 residues 25–200. (D) rhesIE1 residues 215–393 and humIE1 residues 200–382. (TIF) [file ppat.1009863.s002.tif]

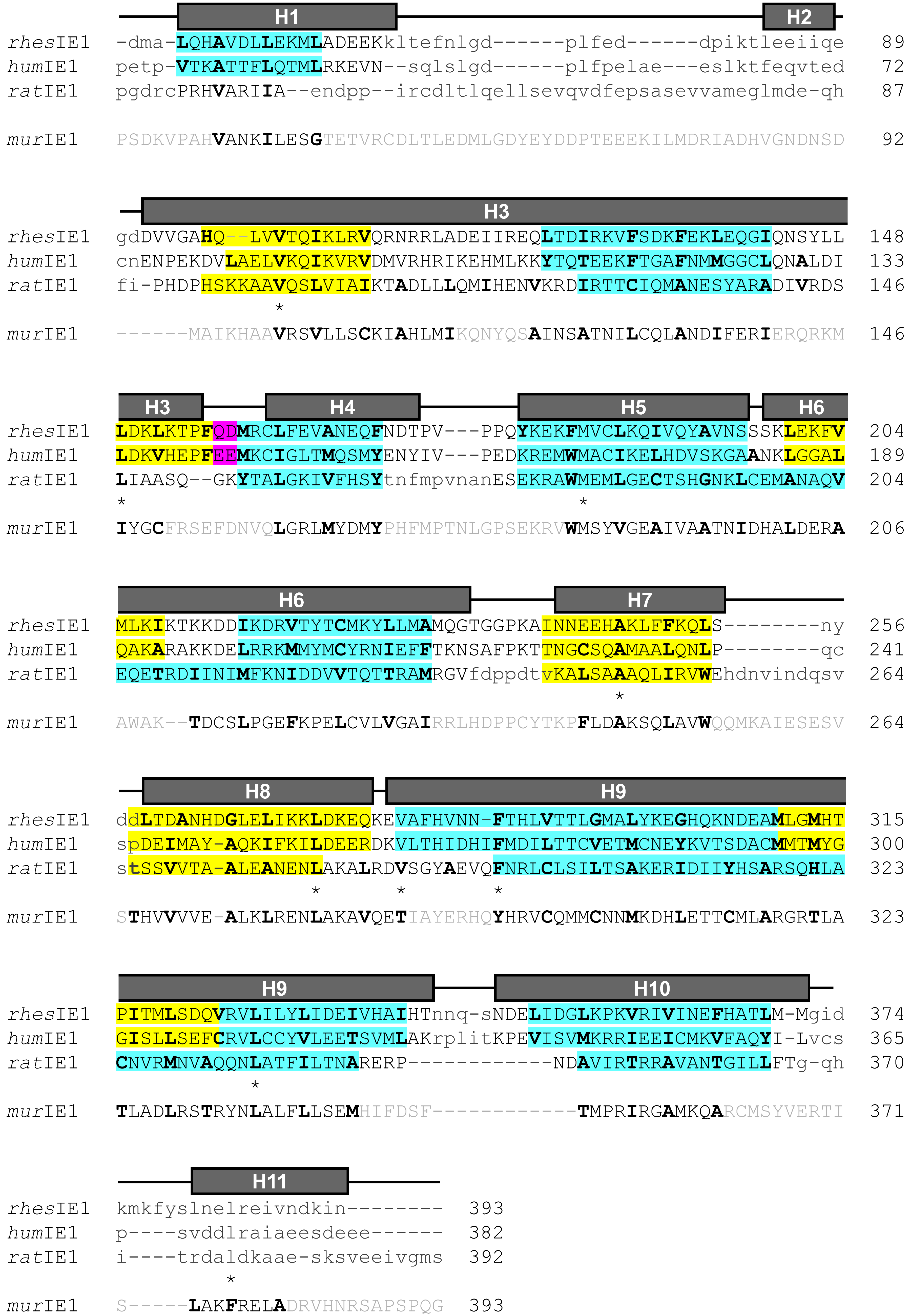

Supplement: S3 Fig — Structure-based sequence alignment calculated with PROMALS3D [32]. The sequences of rhesIE1, humIE1 and ratIE1 were aligned according to the experimental structures. Helix designations were taken from rhesIE1 structure (PDB: 4WID:B). Residues identical in all three structures are marked by an asterisk (*). The handedness of coiled-coils in the structures of rhesIE1, humIE1 and ratIE1 is marked in yellow (left-handed) or cyan (right-handed). The hydrophilic residues of the three-residue insertions are marked in magenta. Residues occupying the a, d or h positions of heptad or hendecad repeats are shown in boldface. Regions without possible repeats are printed in lower case. The sequence of murIE1 was manually fitted to the aligned sequences. Putative residues involved in heptad or hendecad repeats are indicated as described above. (TIF) [file ppat.1009863.s003.tif]

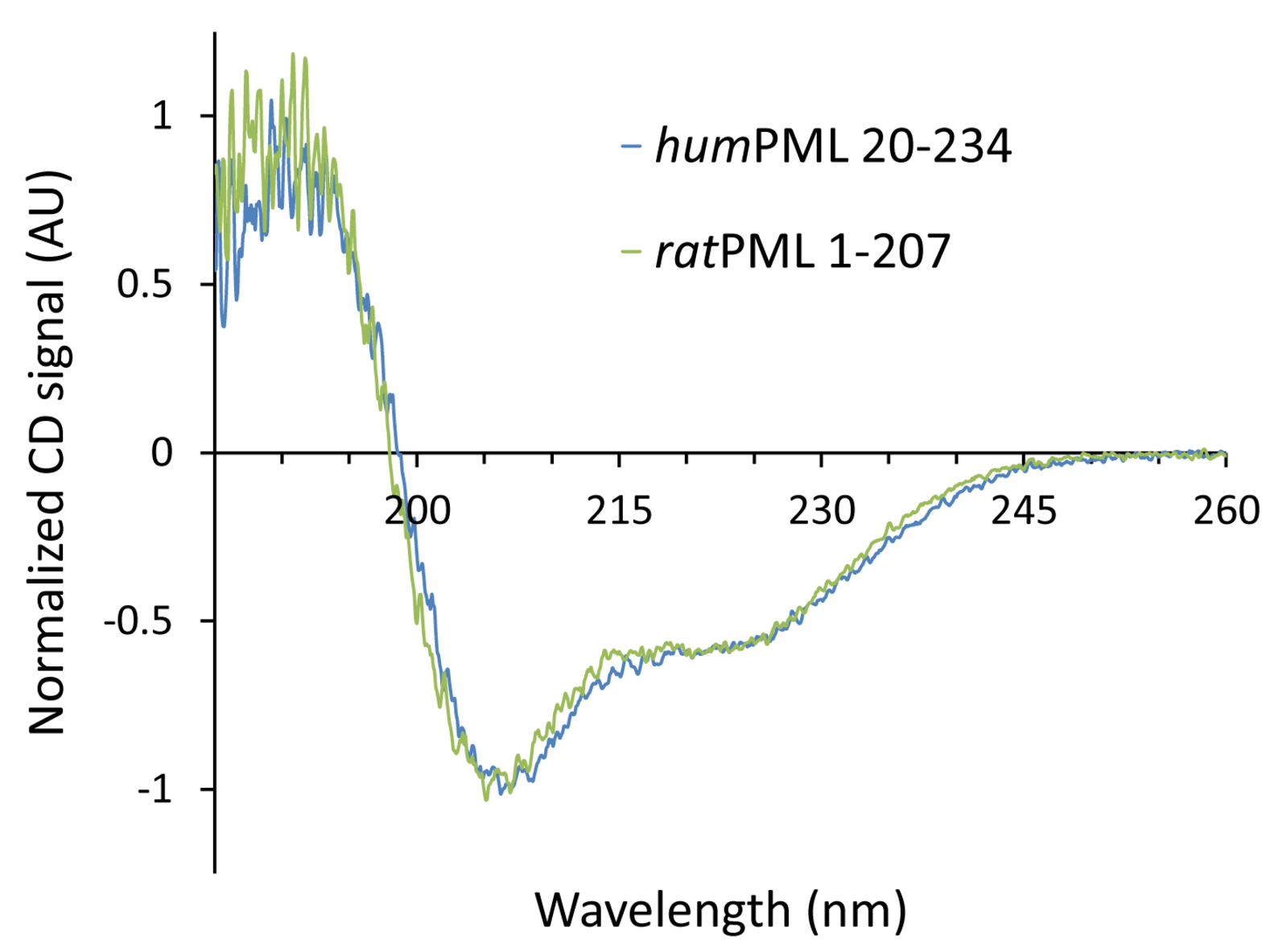

Supplement: S4 Fig — CD spectra of humPML 20–234 (humPML RB) and ratPML 1–207 (ratPML RB). The spectra were normalized at 207 nm as suggested by Raussens and coworkers [57]. The spectra suggest that the humPML and ratPML RB segments share a highly similar secondary structure composition and that both protein variants are properly folded. (TIF) [file ppat.1009863.s004.tif]

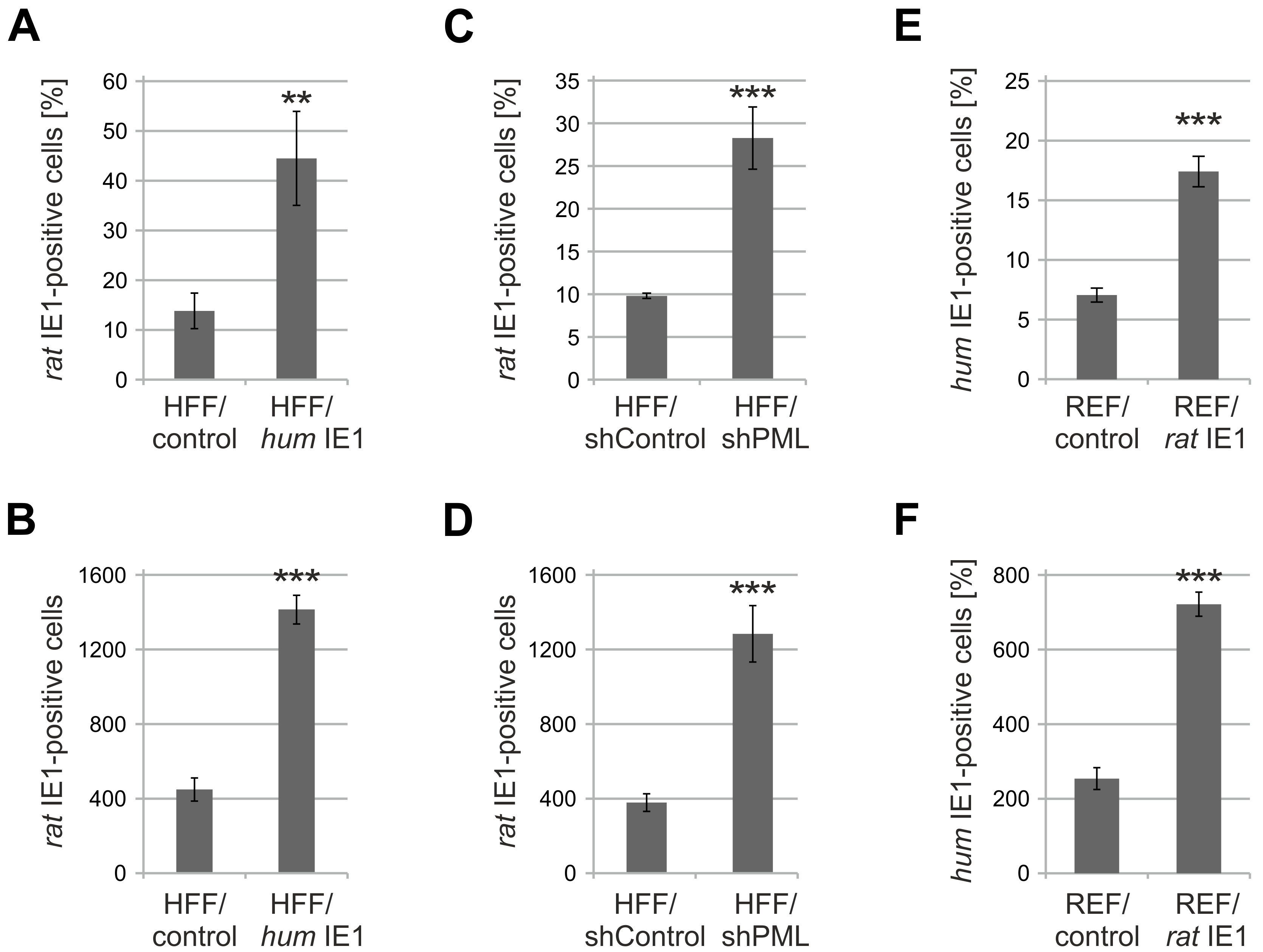

Supplement: S5 Fig — (A, B) HumIE1 expression in HFF enhances RCMV IE gene expression. Control HFF or humIE1-expressing HFF were treated with doxycycline for 24h, followed by RCMV-E infection at an MOI of 0.1 (A) or with a low input of < 500 IE units per well (B). 8 hpi, cells subjected to immunofluorescence staining of ratIE1 in order to determine the initiation of lytic gene expression. (C, D) PML depletion from HFF enhances RCMV IE gene expression. Control HFF or PML-knockdown HFF were infected with RCMV-E at an MOI of 0.1 (C) or with a low input of < 500 IE units per well (D). 8 hpi, cells subjected to immunofluorescence staining of ratIE1 in order to determine the initiation of lytic gene expression. (E, F) RatIE1 expression in REF enhances HCMV IE gene expression. Control REF or ratIE1-expressing REF were infected with HCMV strain AD169 at an MOI of 0.1 (E) or with a low input of 250 IE units per well (F). 24 hpi, cells were subjected to immunofluorescence staining of humIE1 in order to determine the initiation of lytic gene expression. All values are derived from triplicate samples and represent mean values ± SD. P-values were calculated using two-tailed Student’s t-test. **, p ≤ 0.01; ***, p ≤ 0.001. (TIF) [file ppat.1009863.s005.tif]
