# Supplementary material for: Cytomegalovirus immediate-early 1 proteins form a structurally distinct protein class with adaptations determining cross-species barriers
Source: PLoS Pathog. 2021 Aug 9;17(8):e1009863. doi: 10.1371/journal.ppat.1009863 (PMC8376021; doi:10.1371/journal.ppat.1009863)
Supplement: S1 Table — (DOCX) [file ppat.1009863.s006.docx]

## S1 Table. Peak assignment in an anomalous difference map calculated with the selenomethionine peak data.

| Peak | Peak height  (intensity/standard deviation) | Coordinates (Å) | | | Se-atom of SeMet residue |
| --- | --- | --- | --- | --- | --- |
| 1 | 16.6 | 83.8 | 62.6 | 20.5 | 215 |
| 2 | 14.6 | 70.3 | 66.9 | 37.3 | 230 |
| 3 | 13.9 | 50.0 | 69.7 | 42.9 | 116 |
| 4 | 13.9 | 75.3 | 66.8 | 30.9 | 132 |
| 5 | 13.8 | 120.3 | 61.0 | -8.0 | 170, 183^a^ |
| 6 | 12.8 | 101.5 | 46.2 | 6.3 | 199 |
| 7 | 12.6 | 118.0 | 51.3 | -4.5 | 185 |
| 8 | 11.9 | 100.5 | 54.0 | 12.0 | 328 |
| 9 | 9.7 | 37.7 | 73.7 | 55.0 | 79 |
| 10 | 7.4 | 43.3 | 82.3 | 57.0 | 83 |
| 11^b^ | 4.6 | 100.4 | 32.3 | 3.4 | 391 |

^a^ Electron density peak extends over both selenium atoms.

^b^ Subsequent electron density peaks could not be assigned.
