# Supplementary material for: Cytomegalovirus immediate-early 1 proteins form a structurally distinct protein class with adaptations determining cross-species barriers
Source: PLoS Pathog. 2021 Aug 9;17(8):e1009863. doi: 10.1371/journal.ppat.1009863 (PMC8376021; doi:10.1371/journal.ppat.1009863)
Supplement: S2 Table — (DOCX) [file ppat.1009863.s007.docx]

## S2 Table. Peak assignment in an anomalous difference map calculated with a long-wavelength data set (2.0 Å, 6 keV).

| Peak | Peak height  (intensity/standard deviation) | Coordinates (Å) | | | Residue and atom type |
| --- | --- | --- | --- | --- | --- |
| 1 | 8.6 | 69.9 | 68.0 | 39.9 | C299 Sγ, M230 Sδ^a^ |
| 2 | 8.4 | 69.9 | 65.1 | 35.2 | C129 Sγ, M230 Sδ^a^ |
| 3 | 8.4 | 115.1 | 50.2 | -2.1 | M185 Sδ, C189 Sγ^a^ |
| 4 | 7.5 | 83.4 | 62.5 | 20.8 | M215 Sδ |
| 5 | 7.4 | 75.5 | 66.5 | 31.0 | M132 Sδ |
| 6 | 7.2 | 101.1 | 53.7 | 11.9 | M328 Sδ |
| 7 | 6.5 | 95.8 | 56.3 | 16.5 | C324 Sγ |
| 8 | 6.5 | 100.8 | 46.2 | 6.2 | M199 Sδ |
| 9 | 6.4 | 103.1 | 55.1 | 4.1 | C197 Sγ |
| 10 | 6.0 | 37.2 | 72.3 | 56.1 | M79 Sδ |
| 11 | 5.8 | 50.8 | 69.7 | 43.0 | M116 Sδ |
| 12 | 5.5 | 68.8 | 59.2 | 26.6 | C37 Sγ |
| 13 | 5.0 | 119.3 | 59.9 | -8.3 | M170 Sδ, M183 Sδ^a^ |
| 14^b^ | 4.4 | 62.0 | 59.3 | 53.1 | C54 Sγ |

^a^ Electron density peak extends over both atoms.

^b^ Subsequent electron density peaks could not be assigned.
