# Supplementary material for: Cytomegalovirus immediate-early 1 proteins form a structurally distinct protein class with adaptations determining cross-species barriers
Source: PLoS Pathog. 2021 Aug 9;17(8):e1009863. doi: 10.1371/journal.ppat.1009863 (PMC8376021; doi:10.1371/journal.ppat.1009863)
Supplement: S3 Table — (DOCX) [file ppat.1009863.s008.docx]

## S3 Table. *Hum*IE1_CORE_ and *rat*IE1_CORE_ homologous protein structures as identified using the Dali webserver^a^.

| Rank^b^ | Protein name | PDB-  entry | Z-Score | RMSD (Å)^c^ | Nr. of aligned residues/ total nr. of residues present in the deposited model | Identity (%) |
| --- | --- | --- | --- | --- | --- | --- |
| Query: *hum*IE1_CORE_ (6TGZ, 352 residues) | | | | | | |
| 1 | *rhes*IE1_CORE_ | 4wid | 28.0 | 2.3 | 337/353 | 22 |
| 2 | *rat*IE1_CORE_ | 6th1 | 20.1 | 4.6 | 319/360 | 9 |
| 3 | Sporozoite microneme protein essential for cell traversal | 4u5a | 11.0 | 3.0 | 137/172 | 7 |
| 4 | Three helix bundle (designed) | 4tql | 10.1 | 9.5 | 225/235 | 10 |
| 5 | Inhibitor of nuclear factor kappa-B kinase subunit alpha | 5ebz | 9.9 | 9.5 | 235/655 | 9 |
| 6 | ATG29 | 4p1w | 9.8 | 10.6 | 238/389 | 6 |
| 7 | Serine/threonine-protein kinase TBK1 | 4iwp | 9.7 | 9.9 | 235/628 | 9 |
| Query: *rat*IE1_CORE_ (6TH1, 360 residues) | | | | | | |
| 1 | *hum*IE1_CORE_ | 6tgz | 20.1 | 4.6 | 319/352 | 9 |
| 2 | *rhes*IE1_CORE_ | 4wid | 18.3 | 4.0 | 316/353 | 9 |
| 3 | Inhibitor of nuclear factor kappa-B kinase subunit alpha | 5ebz | 9.7 | 8.8 | 240/655 | 7 |
| 4 | Inhibitor of nuclear factor kappa-B kinase subunit beta | 4kik | 9.5 | 9.1 | 220/618 | 10 |
| 5 | Three helix bundle (designed) | 4tql | 9.3 | 9.0 | 224/235 | 9 |
| 6 | Serine/threonine-protein kinase TBK1 | 4jl9 | 9.3 | 9.6 | 236/646 | 4 |
| 7 | ATG29 | 4p1w | 9.2 | 11.2 | 237/399 | 6 |

^a^ Ref([1,2])

^b^ In cases where different databank entries described the same protein or contained multiple copies of the same protein, the results are listed for the copy/entry yielding the highest Z-score only.

^c^ Calculated using Cα atom positions.

## Supplementary references

1. Holm L, Laakso LM. Dali server update. Nucleic Acids Research. 2016; 44(W1):W351-W5. https://doi.org/10.1093/nar/gkw357 PMID: 27131377.

2. Rose PW, Prlic A, Altunkaya A, Bi C, Bradley AR, Christie CH, et al. The RCSB protein data bank: integrative view of protein, gene and 3D structural information. Nucleic Acids Res. 2017; 45(D1):D271-D81. https://doi.org/10.1093/nar/gkw1000 PMID: 27794042.
