# Supplementary material for: Cytomegalovirus immediate-early 1 proteins form a structurally distinct protein class with adaptations determining cross-species barriers
Source: PLoS Pathog. 2021 Aug 9;17(8):e1009863. doi: 10.1371/journal.ppat.1009863 (PMC8376021; doi:10.1371/journal.ppat.1009863)
Supplement: S4 Table — (DOCX) [file ppat.1009863.s009.docx]

## S4 Table. Oligonucleotides

| Oligonucleotides for cloning of RCMV IE1 (*rat*IE1) versions into prokaryotic expression plasmid pGEX-6-P1 | |
| --- | --- |
| 5'rIE1co_BamHI | CATAGGATCCATGGACCCGACCCTGTTTAC |
| 5'rIE1co_aa30_BamHI | CATAGGATCCGAACAGCAGCCTGGTGATCG |
| 3'rIE1co_SalI | CATAGTCGACTTAACTACGACGACCACG |
| 3'rIE1co_aa392_SalI | CATAGTCGACTTAGCTCATACCAACAATTTCTTC |
| Oligonucleotides for cloning of *hum*PML versions into prokaryotic expression plasmid pGEX-6-P1 | |
| PMLoN20_6P | CGCGGATCCGAACCGACCATGCCTCCGCC |
| PMLoC255 | CCGCTCGAGTTAACCAAATGCGCTATCCTGTTCTTGCAG |
| PMLo_Q235stop_for | atatttcagccgaaattcagtaacgtcaagaggaactggatgc |
| PMLo_Q235stop_rev | gcatccagttcctcttgacgttactgaatttcggctgaaatat |
| Oligonucleotides for cloning of ratIE1 and ratPML variants into eukaryotic expression plasmids pHM971 and pHM1580 | |
| 5'BglII_rat IE1_aa2 | CATAAGATCTGATCCGACCCTGTTTAC |
| 3'EcoRI_rat IE1_aa565 | CATAGAATTCTCAAGAGCGACGACCACG |
| 3'EcoRI_rat IE1_aa392 | CATAGAATTCTCAGGACATGCCCACAATC |
| 5'rPML-start2-BamHI | CATAGGATCCCCTCCCCCAGAGGAACCCTC |
| 5'rPML-aa94-BamHI | CATAGGATCCCGGCAGATTGTGGATGCG |
| 3'rPML-EcoRI | CATAGAATTCTTAGGCCAGGCATCCCTTATTTTC |
| 3'rPML-aa405-EcoRI | CATAGAATTCTTATGGCTGAGCGCTGGCTGCC |
| 3'rPML-aa207-EcoRI | CATAGAATTCTTACTGGATCTCCTTACCAATTTCACACTGG |
| Oligonucleotides for cloning of HCMV IE1 (humIE1) variants into eukaryotic expression plasmid pInducer20 | |
| c-CRS-mut | GCGTGTACGGTGGGAGGCCTATATAAGCAGAGCCTAGGTAGGGAGAAGTCAGATCGCCTGGAGACGCC |
| nc-CRS-mut | GGCGTCTCCAGGCGATCTGACTTCTCCCTACCTAGGCTCTGCTTATATAGGCCTCCCACCGTACACGC |
| 5'attB1_FLAG | GGGGACAAGTTTGTACAAAAAAGCAGGCTATGGACTACAAAGACGATGA |
| 3'attB2_IE1 | GGGGACCACTTTGTACAAGAAAGCTGGGTCTTACTGGTCAGCCTTGCTTC |
| 3'rIE1_attB2 | GGGGACCACTTTGTACAAGAAAGCTGGGTCTCAAGAGCGACGACCACGTTTAG |
| Oligonucleotides for real-time PCR | |
| 5'RCMV-gB | CAAGGCCATCAGAACGGATC |
| 3'RCMV-gB | TCACTCCCCGATGCGTTATA |
| RCMV-gB FAM/TAMRA | TCACTCCCCGATGCGTTATA |
| SiRNA target sequences | |
| siPML2 | AGATGCAGCTGTATCCAAG |
| siC | GTGCGTTGCTAGTACCAAC |
